# Effectiveness of a pharmacist-led, community group-based education programme in enhancing diabetes management: A multicentre randomised control trial

**DOI:** 10.1016/j.conctc.2024.101280

**Published:** 2024-02-24

**Authors:** Kamarudin Ahmad, Lawrence Anchah, Chuo Yew Ting, Su Ee Lim

**Affiliations:** a Miri Hospital and Clinical Research Center Miri, Miri, Sarawak, Malaysia; b Pharmacology Unit, Faculty of Medicine and Health Sciences, University Malaysia Sarawak, Malaysia; c Pharmacy Practice and Development, Pharmacy Service Division, Sarawak State Health Department, Malaysia

**Keywords:** Pharmacy integrated community care (PICC), Type 2 diabetes mellitus (T2DM), Hemoglobin A1c (HbA1c), Medication adherence, Diabetes education program

## Abstract

**Aims:**

This study presents a protocol for the Pharmacy Integrated Community Care (PICC) program, meticulously designed to enhance Hemoglobin A1c (HbA1c) levels and augment knowledge about diabetes mellitus (DM) among individuals diagnosed with Type 2 Diabetes Mellitus (T2DM) in the Sarawak State of Malaysia.

**Methods:**

From 1 May to December 31, 2023, a prospective, multicenter, parallel-design randomised controlled trial will be conducted with two groups, each consisting of 47 participants. The intervention group will receive a structured, four-session group-based program guided by experienced pharmacists, focusing on medication adherence and diabetes management. The control group will follow the standard Diabetes Mellitus Adherence Clinic program. The primary outcomes of this study encompass enhancements in knowledge regarding diabetes medication management and adherence, followed by subsequent changes in HbA1c levels.

**Conclusions:**

The successful implementation of the PICC program holds promise for enhancing health outcomes in the T2DM population, potentially leading to more effective diabetes management initiatives and better health practices in the community.

**Trial registration clinicaltrials.gov identifier:**

NCT05106231.

## Highlights

• Despite efforts, medication adherence remains challenging in DM management.
• Group-based interventions (GBIs) potentially improve adherence and knowledge.
• PICC protocol will investigate adherence, knowledge and HBA1C in DM patients.
• The study employs a rigorous trial, comparing PICC to standard DMTAC.
• Findings could shape more effective diabetes management.

## 1 Introduction

Diabetes mellitus (DM) constitutes a significant global health challenge, with its prevalence surging from 4.7% in 1980 to 8.5% in 2014 [1]. Notably, in Malaysia, the pooled diabetes prevalence spanning from 1995 to 2021 has reached 14.39% [2]. Addressing this escalating health crisis, Malaysia has implemented several strategic initiatives, including the National Plan of Action for Nutrition (NPAN III) (2016–2025) and the National Strategic Plan for Non-Communicable Disease (2010–2014) [3,4].

Despite comprehensive efforts in primary and tertiary care [5], non-adherence to DM medication therapy remains a challenge, with rates ranging from 36% to 93% [6,7]. Local research on patient satisfaction and medication adherence among type 2 diabetes mellitus (T2DM) patients is limited [8,9], underscoring the need for innovative interventions.

In response to this, ‘Know Your Medicine – Take it for Health’ (MEDIHEALTH) and similar to group-based interventions (GBIs) have shown promise in improving medication adherence with T2DM [[10], [11], [12]]. In 2020, Malaysia introduced the innovative Pharmacy Integrated Community Care (PICC) program, which distinguishes itself with the incorporation of trained civilian Ambassadors of Know Your Medicine (AKYM), which play a crucial role, serving as vital bridges within PICC [13].GBIs, including PICC, involve regular sessions covering various aspects of diabetes management and foster collaboration between facilitators and healthcare professionals [14,15]. The success of these interventions is grounded in the Social Cognitive Theory (SCT) and Health Belief Model (HBM), emphasising observational learning, social support, self-efficacy, and feedback in diabetes management [[16], [17], [18], [19]].

Pharmacy Integrated Community Care (PICC) distinguishes itself through its innovative incorporation of trained civilian Ambassadors of Know Your Medicine (AKYM), playing a crucial role in recruitment and providing vital peer support [20]. PICC collaborates seamlessly with diverse healthcare professionals, including pharmacists, dietetic officers, nurses, medical officers, and physiotherapists, propelling it to participant-centric and holistic care [20]. AKYM ambassadors, pivotal to PICC's structure, contribute significantly to recruitment by leveraging pre-existing community relationships, acting as intermediaries to establish bonds and facilitate a personalised learning process [20]. This approach enhances participant motivation and engagement, underscoring the program's effectiveness in fostering a supportive and community-oriented environment for diabetes management [20].

A randomised controlled trial (RCT) is being conducted to assess the effectiveness of PICC, driven by the imperative for a rigorous scientific approach to establish a causal relationship and overcome biases associated with observational studies [21]. Despite existing evidence, including the success of MEDIHEALTH, highlighting the potential of various GBIs in improving medication adherence [[10], [11], [12],^22^], the uniqueness of PICC, especially its incorporation of Ambassadors of Know Your Medicine (AKYM), necessitates a focused investigation. While ample evidence compares the effectiveness of different GBIs [23], PICC stands out due to its innovative approach. The RCT design compares PICC and the established gold standard of care in Malaysia, the Diabetes Medication Therapy Adherence Clinic (DMTAC) [21]. This design thoroughly evaluates PICC's impact on medication adherence, knowledge, and HbA1C levels [21].

The theoretical foundation of PICC posits it as an independent determinant capable of enhancing the model and positively impacting HbA1C levels [23]. Building on the Social Cognitive Theory (SCT) and Health Belief Model (HBM), PICC emphasises observational learning, social support, self-efficacy, and feedback in diabetes management [[17], [18], [19]]. In addition to this, the study's conceptual framework generates specific hypotheses:

1. There is a significant difference in medication adherence to diabetes medication between the PICC group compared to the control group.
2. There is a significant difference in the reduction of HbA1C between the PICC group and the control group with higher medication adherence.
3. There is a significant difference in knowledge of diabetes medication between the PICC group compared to the control group.
4. There is a significant difference in the reduction of HbA1C between the PICC group and the control group, which has a higher understanding and knowledge of diabetes.

The tools utilised in this study are the Diabetes Knowledge Test (Modified DKT) and the Malaysia Medication Adherence Assessment Tool (MyMAAT) to assess observational learning, self-efficacy, and knowledge [24,25]. These assessments provide insights into patients' experiences, social support networks, self-efficacy beliefs, and knowledge related to diabetes management, informing the development of interventions [26].

PICC protocol aims to assess the impact of the PICC program on medication adherence, knowledge, and HbA1C levels in T2DM patients through randomised controlled trials, with one group undergoing the intervention (PICC group) and the other serving as a control (DMTAC group). The findings will provide valuable insights into improving medication adherence, knowledge, and HbA1C levels among Malaysian T2DM patients.

## 2 Methods

### 2.1 Trial design

PICC trials adopt a prospective, multicenter, parallel-design randomised controlled trial featuring two treatment groups. The protocol aligns with the Standard Protocol Items: Recommendations for Interventional Trials (SPIRIT) checklist [27]. The trial assesses the efficacy of a Pharmacy Integrated Community Care (PICC) intervention in lowering HbA1C levels. The intervention involves four two to 3-h sessions of group-based intervention (GBI) led by pharmacists, with follow-up evaluations. The control group receives standard treatment, Diabetes Medication Therapy Adherence Clinic (DMTAC). The trial period spans from 1 May to December 31, 2023.

### 2.2 Sample size

**Fig. 1:**
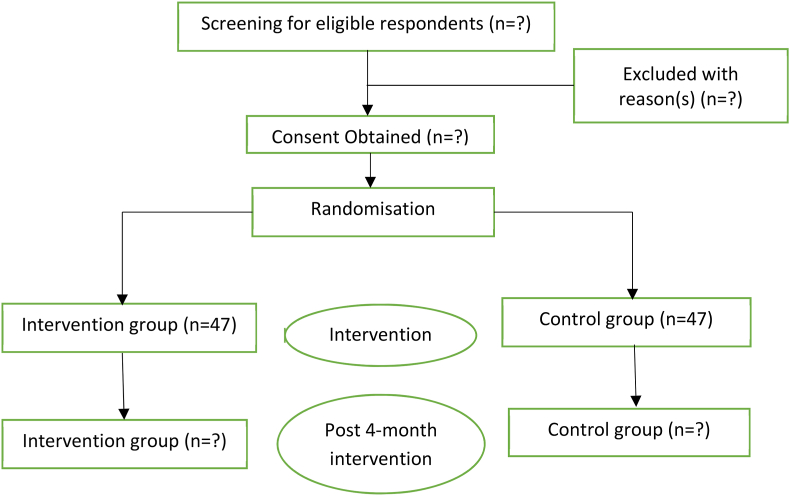
Trial flow following consolidated standards of reporting trials (CONSORT).

PICC trials involve a continuous response variable from independent and experimental subjects, with one control per experimental subject. In a previous study, each subject group's response was normally distributed with a standard deviation 1.5 [11]. If the actual difference between the experimental and control means is 1, 36 experimental and 36 control subjects would be required to reject the null hypothesis that the population means of the experimental and control groups are equal, with a probability (power) of 0.8. By factoring in an estimated 30% dropout or incomplete data, a minimum sample size of 94 (47 for each group) was pre-determined, as depicted in Fig. 1.

### 2.3 Study site and recruitment

The PICC trials encompassed several primary government health clinics in each district in Sarawak, strategically orchestrating pharmacy-integrated community care (PICC) sessions at these locations. Participants were recruited through consecutive sampling during routine visits from 1 May to December 31, 2023, involving comprehensive briefings about the study and extending invitations to interested individuals. Employing a random allocation process ensured impartial assignment, with participants assigned to either the PICC treatment group or the control group (Diabetes Medication Therapy Adherence Clinic - DMTAC). The scheduling of multiple PICC sessions was meticulously planned, with participants informed about specific dates during routine visits before the intervention. Notably, participants in the PICC group were not offered direct incentives or compensation for transportation, thus preserving the real-world applicability of the intervention. The involvement of Ambassadors of Know Your Medicine (AKYM) in recruitment capitalises on pre-existing community relationships, emphasising a personalised approach to enhance participant engagement and motivation.

### 2.4 Inclusion criteria

Eligible participants for this study were non-pregnant adults aged >18 years, irrespective of gender or ethnicity, who spoke and understood Bahasa Malaysia. In addition, potential participants were required to have a medical record indicating a haemoglobin A1c test (HbA1c) level of ≥6.0% (42 mmol/mol) and a fasting plasma glucose test (FPG) ≥7.0 mmol/L. Furthermore, individuals had to demonstrate the ability to provide informed consent to participate in the study.

### 2.5 Exclusion criteria

Participants were excluded if they could not answer the quizzes independently or had impairments in hearing or vision. Additionally, individuals who could not read, write, and speak Malay and those deemed medically unstable or incapable of providing informed consent were excluded from the study. Patients attending intensive psychological treatment, hospitalised, or participating in other studies were also excluded.

### 2.6 Extraneous variables/confounding variables

Extraneous variables encompass dimensions from literature, including patient-related, condition-related, socioeconomic, health system-related, and therapy-related factors. Recent reviews categorise variables as patient, prescription, and prescriber factors [28,29]. Variables incorporated in the study comprise route of administration, number and frequency of medications, age, gender, education, income, employment status, complications, traditional medicines, residential area, social support, diabetic education, and enrollment in diabetic DMTAC.

### 2.7 Randomisation and allocation concealment

Participants who learn from pharmacists about PICC and DMTAC during routine visits preceding the intervention and through AKYM are invited to participate. Those allocated to the PICC group receive comprehensive program details in Appendix 1 and specific attendance dates. Control group participants are scheduled for DMTAC appointments. Allocation concealment ensures participant confidentiality and patient information is coded for secure storage.

The Sarawak Pharmacy Service Division conducts a simple randomisation process using an online program at http://www.graphpad.com/quickcalcs/index.cfm. An appointed officer manages the process, ensuring participant anonymity through unique codes. Group assignments (intervention or control) are maintained securely by the Division to uphold blinding.

Participants receive appointment dates without knowledge of their group assignment. After participant arrival, facilitators learn of the assigned activity (intervention or control), preserving blinding.

Post-intervention, researchers remain blinded as the Sarawak Pharmacy Service Division retains the participant list with group assignments. The unblinding occurs only after the study publication.

Notably, the educational nature of the study eliminates the need for code-breaking. In serious adverse events, the principal investigator reports to the Medical Research and Ethics Committee, with code-breaking occurring only after ruling out study-related causation. This robust process ensures ethical conduct and participant safety.

### 2.8 Intervention

The study incorporates the Pharmacy Integrated Community Care (PICC), a Group-Based Intervention (GBI) developed by Malaysia's Pharmaceutical Services Division. PICC is designed to complement individualised approaches by improving the understanding of medication management for Type 2 Diabetes Mellitus (T2DM), as shown in Appendix 1. This method reaches a broader patient base, efficiently fostering crucial medication adherence skills compared to individual strategies. GBIs, such as PICC, offer advantages such as validation, normalisation, reduced isolation, a sense of belonging, and heightened self-esteem [30]. PICC sessions, conducted by one principal and three assistant facilitators, extend over two to 3 h each and encompass four consecutive monthly modules. In contrast, the control group receives a 3-h lecture covering the same syllabus but without the interactive elements of a GBI.

The study evaluates the effectiveness of PICC through laboratory-analysed HbA1C levels, the Diabetes Knowledge Test (Modified DKT), and the Malaysia Medication Adherence Assessment Tool (MyMAAT), measured during the initial and fourth visits. This comprehensive assessment approach ensures a thorough understanding of the impact of the intervention on glycemic control, diabetes knowledge, and medication adherence for the study. It is important to note that the PICC module strictly adheres to the guidelines outlined in the GUIDED Checklist [31], as illustrated in Table 1, ensuring the intervention's alignment with established standards and best practices for optimal research integrity.

**Table 1:** GUIDED Checklist for reporting intervention development studies in health research.

GUIDE items	Study compliance
Report the purpose of the intervention.	PICC program investigates medication adherence, knowledge, and HBA1C levels in Sarawak residents with type 2 diabetes mellitus (T2DM) through randomized controlled trials (RCTs).
Report the target population.	The intervention targets residents of Sarawak with diverse backgrounds and T2DM, recruited from primary government health clinics, ensuring a representative sample for comprehensive analysis.
Report any use of components from an existing intervention	Elements from ‘Know Your Medicine – Take it for Health’ (MEDIHEALTH) and similar group-based interventions (GBIs) are incorporated into the PICC program, leveraging successful elements from established interventions.
Report how evidence from different sources informed the intervention development.	Informed by robust assessments, including the diabetes knowledge test (Modified DKT) and Malaysia medication adherence assessment tool (MYMAAT), ensuring a comprehensive understanding of the intervention's potential impact.
Report how stakeholders contributed to the intervention development process.	Stakeholder contributions include insights from trained facilitators and qualitative patient interviews, providing diverse perspectives that enrich the development process.
Report important uncertainties at the end of the intervention development process.	Noteworthy uncertainties include the potential for selection bias, the relatively short follow-up period, and limitations in assessing long-term outcomes, acknowledging potential influences on the study's conclusions.
Report the context in which the intervention was developed	Addressing the escalating prevalence of diabetes mellitus in Malaysia, the PICC program focuses specifically on non-adherence to DM medication therapy in T2DM patients, addressing a critical aspect of diabetes management.
Report any changes to interventions required or likely to be required for subgroups.	No adjustments to the intervention are deemed necessary for subgroups; participant selection follows explicit inclusion and exclusion criteria, ensuring a consistent approach across the study population.
Report how any published intervention development approach contributed to the development process.	The intervention aligns with established standards, following the guidelines outlined in the standard protocol items: recommendations for interventional trials (SPIRIT) checklist, ensuring a rigorous and systematic development process.
Report how existing published theory informed the intervention development process.	Grounded in the social cognitive theory (SCT) and health belief model (HBM), the PICC program leverages established theories to inform its development, providing a theoretical framework for understanding and modifying health-related behaviours.
Report any guiding principles, people, or factors prioritized when making decisions.	Decision-making prioritizes a comprehensive evaluation of the PICC program's impact through a randomized controlled trial (RCT) design, ensuring rigorous scrutiny of the intervention's efficacy.
Report how the intervention changed in content and format from the start of the intervention development process.	The structured PICC program unfolds over four weeks with distinct sessions focused on critical aspects of diabetes management. In the initial week, the program aims to cultivate a support group to enhance patients' self-motivation, guiding participants to recognize and understand diabetes while fostering information sharing. Subsequent weeks delve into practical aspects, emphasising exposure to the diet and lifestyle of individuals with diabetes, understanding antidiabetic medications, and recognizing complications of diabetes. Each session facilitates information sharing among participants, reflecting an iterative development process based on feedback and insights to optimize content and format.
Report the reasons for discarding intervention components that were considered.	No discarding intervention components will be considered, ensuring the integrity and continuity of the PICC program.
Follow TIDIer guidance when describing the developed intervention	Detailed descriptions, following TIDIer guidance, are provided in the document's main body, outlining the PICC program's components, procedures, and other essential aspects for transparency and replicability.
Report the intervention development in an open-access format	Comprehensive study details, fostering transparency and collaboration among researchers and the public, are accessible through protocol NCT05106231 on clinicaltrials.gov, ensuring an open-access approach to disseminating study information.
Report the background and contribution of those making decisions about the intervention content, format, and delivery.	Decision-makers, led by Mr. Mohd. Shafiq Yusuf and his team participated in the innovative and creative group competition in 2020, providing insights and innovations that shaped the development of the PICC program.
Report the time taken to develop the intervention.	The PICC program was designed within a year and initiated by Malaysia's Innovative and Creativity Group, showcasing efficiency and timeliness in the development process.
Report who, when why and where the original idea for developing the intervention came from	The original idea for the PICC program originated from Mr. Mohd. Shafiq Yusuf and his team, participants in the innovative and creative group competition in 2020, highlighted the intervention's creative genesis.

Furthermore, it is essential to highlight the unique aspect of the PICC as a GBI. What sets PICC apart from other initiatives is the integral role played by Ambassadors from Know Your Medicine (AKYM), who assist in this program. AKYM Ambassadors are instrumental in the recruitment process, leveraging their deep-rooted connections within the community to identify and engage participants. AKYM Ambassadors utilise their established rapport to foster trust and facilitate communication as intermediaries between pharmacists and the community. The personalised approach enhances recruitment and contributes to a smoother learning experience within the PICC program. Already acquainted with AKYM Ambassadors, participants exhibit heightened motivation and enthusiasm, ultimately fostering greater success and engagement in the program.

### 2.9 Facilitators

Facilitators undergo training and demonstrate intervention alignment three sessions before the study's commencement. Their efficacy is evaluated based on educational handling, procedure adherence, participant interaction, and responsiveness. Each structured group-based intervention session involves one primary and three assistant facilitators, accommodating 5 to 10 participants. All facilitators are pharmacists under the Sarawak State Health Department's Pharmaceutical Services Division.

### 2.10 Control group content

The control group undergoes standard treatment through the Diabetes Medication Therapy Adherence Clinic (DMTAC), as delineated in Appendix 2. DMTAC Clinics, administered by trained pharmacists in DMTAC at health clinics, involve baseline HbA1C assessment on the initial appointment date and a subsequent evaluation during the fourth session, concluding this phase of service. DMTAC participants strive to attend monthly sessions, and flexibility in appointment adjustments is permitted, distinguishing it from the PICC group. The one-to-one sessions with the pharmacist afford participants a personalised experience. Both intervention and control group participants must fulfil the comprehensive four-session program requirements. This flexibility ensures robust participant engagement while upholding the study protocol's integrity, fostering a nuanced understanding of each participant's journey within the study.

In the context of DMTAC, participants experience an encompassing follow-up spanning a minimum of four visits, incorporating various essential interventions. These include meticulous medication adherence assessment, adept identification and management of drug-related issues, targeted medication counseling, rigorous monitoring of clinical outcomes, and methodical diabetes education delivered by the assigned pharmacist. This multifaceted approach guarantees that control group participants receive thorough and personalised care throughout the study, aligning with the study's objectives and contributing to the overall effectiveness of the intervention.

Comprehensive guidelines for effective diabetes management are provided to DMTAC participants. For a detailed overview of the materials distributed during DMTAC sessions, please refer to Appendix 2**.**

### 2.11 Case Report Form (CRF)

The Case Report Form (CRF), initially drafted in Bahasa Malaysia, served as a comprehensive tool for data collection. The myPICC Activity Book, inclusive of module quizzes, and the Record Book PICC were utilised to record essential patient data, encompassing HbA1C levels, medication history, and referrals. Additionally, the CRF integrated two validated Malay questionnaires: MyMAAT and Modified DKT [24,25]. The administration of the CRF was conducted by trained facilitators who were part of the study team. Facilitators filled in the relevant sections of the CRF based on the information provided by the participants during the study sessions, ensuring accuracy and consistency in data collection.

### 2.12 Treatment fidelity

The assessment of treatment adherence for the GBI will follow the methods and principles established by the Treatment Fidelity Workgroup associated with the Behaviour Change Consortium of the National Institutes of Health [32]. The framework of treatment fidelity strategies for this study is outlined in Table 2.

**Table 2:** Framework of treatment fidelity strategies.

Components	Goal	Strategies
Study design	Maintain consistent treatment doses within and across conditions.	The GBI intervention protocol is structured to comprise four sessions. Facilitators will adhere to the provided intervention manual for consistent delivery. Researchers will observe a mock intervention by facilitators to ensure delivery appropriateness, provide feedback post-observation, and address any issues. Strict adherence to the allocated time for each activity is mandatory for all facilitators.
	Plan for implementation setbacks.	All staff members in the studied facilities have received training to facilitate the GBI intervention. This training equips them with the skills needed to conduct the intervention independently, even without the initial facilitators. This contingency plan ensures the intervention's uninterrupted progress, even in unforeseen circumstances.
Provider training	Standardise training.	The study will employ a team of skilled facilitators for the GBI intervention. These facilitators will receive joint training to maintain consistent delivery of the intervention. Researchers will observe mock interventions during training sessions conducted by the involved facilitators to evaluate their performance. This observation aims to provide feedback, identify areas for improvement, and ensure that the intervention adheres to the intended delivery standards.
	Ensure provider skill acquisition.	Standardised patients or pilot participants (role-playing) will be used to ensure effective and consistent intervention implementation. The observation of the implementation process will assess the competency and qualifications of the facilitators involved, allowing for necessary adjustments as needed.
	Minimise “drift” in provider skills.	Researchers will maintain ongoing monitoring of the intervention conducted during the study to ensure adherence to the protocol and consistency. Following each PICC session, facilitators can discuss any issues they encountered with the intervention during a post-mortem meeting with researchers. Participants must complete a workbook to document the delivery of specific treatment components, which will be a valuable resource for evaluating the intervention.
	Accommodate provider differences.	The study's facilitators will be pharmacists employed at the primary health clinic of the selected study site. All facilitators will possess uniform knowledge and undergo training to ensure consistent program implementation. This homogeneity within the facilitator pool will promote consistent and effective intervention delivery to all participants.
Treatment delivery	Control for provider differences.	To assess the facilitators' effectiveness, participants were asked to rate their satisfaction level after each session through a questionnaire. These ratings were examined following each session. A qualitative interview was conducted after the study to delve deeper into participants' perceptions and collect additional insights.
	Reduce differences within the treatment.	A scripted intervention protocol is made available to facilitators during training, which is then evaluated through a mock intervention to ensure standardisation of delivery.
	Ensure adherence to the treatment protocol.	The module's script is carefully constructed to provide clear and concise instructions for the facilitator. In tandem with this script, a workbook is provided for the facilitator and participants to complete together, serving as both an engaging activity and a means of verifying the content delivered.
	Minimise contamination between conditions	The study randomly assigned participants to the PICC or control group. To guarantee each participant received individualised treatment, they were placed in their respective groups and provided with a comprehensive orientation to the study procedures. The interventions were distinctly defined, with the PICC group participating in group sessions and the DMTAC group having one-on-one sessions.
Treatment receipt	Ensure participant comprehension.	Program participants will receive a workbook featuring session-related questions to facilitate their learning. Any arising misconceptions will be promptly addressed to ensure a clear understanding of the material. After the intervention, researchers will conduct a qualitative interview to evaluate the program's effectiveness and impact.
	Ensure the participant's ability to use cognitive skills.	At the end of each session, the facilitator will review the participant workbooks to verify comprehension of the target content, correcting any misconceptions. Additionally, quizzes will be administered to participants to evaluate their understanding of the material covered during the session.
	Ensure the participant's ability to perform behavioural skills.	Participant's compliance with the program will be evaluated by administering the MYMATT questionnaire in conjunction with HbA1C testing. Pre- and post-intervention testing will measure behaviour change due to the program.
Enactment of treatment skills	Ensure participant use of cognitive skills.	Using quizzes, workbooks, and the Malay version of the Modified Diabetes Knowledge Test (DKT) questionnaire will effectively enhance, stimulate, and evaluate participants' knowledge. These tools offer an engaging and enjoyable way for participants to acquire information and participate in the session.
	Ensure participants use behavioural skills.	Assessing compliance through the MYMAAT questionnaire and monitoring HbA1c levels prove effective for behaviour change evaluation. By evaluating these measures before and after the intervention, researchers can acquire valuable insights into their effectiveness and confirm the desired behavioural changes have taken place.

### 2.13 Qualitative evaluation

This study integrated qualitative interviews with patients undergoing Pharmacy Integrated Community Care (PICC) procedures. Semi-structured interviews, capturing rich narratives, were meticulously recorded, transcribed, and thematically analysed using Excel. Ten participants, purposefully selected by the program's managerial officers, engaged in In-depth interviews conducted six months post-intervention to understand the long-term impact better. These sessions involved only two independent interviewers, not affiliated with the program, ensuring objectivity and openness in the responses. All participants provided informed consent, and the study was conducted at diverse divisional sites to ensure a broad range of perspectives.

The qualitative evaluation involved in-depth interviews to explore personal experiences through open-ended questions. Face validation was conducted using expert judgment to ensure clarity and relevance, and pilot interviews were carried out to refine the process. These in-depth sessions, lasting between 35 and 50 min each, were conducted in private clinic settings. Demographic information was collected before each interview. Following transcription, measures were taken to maintain participant confidentiality and research integrity by securely disposing of anonymised audio recordings.

Data analysis ran concurrently with data collection, ensuring an iterative and comprehensive approach. After each interview, comprehension was summarised, and insights were documented in debriefing sessions. Transcripts were cross-verified with recordings and meticulously analysed using Excel. Coding procedures commenced after a thorough review of the dataset.

A purposive sampling approach guided the selection of 10 active participants engaged in the PICC program, including two facilitators and two managerial officers overseeing the program. The remaining six participants were selected based on their willingness to participate in the interviews, ensuring a diverse representation.

Importantly, interviewees were not acquainted with the facilitators conducting the in-depth interviews; only two independent interviewers, not affiliated with the program, facilitated the sessions, ensuring objectivity and openness in the responses.

Participants in the PICC group were intentionally not offered direct incentives or transportation compensation, a deliberate choice aimed at maintaining the real-world applicability of the intervention.

### 2.14 Study timeline and procedure

**Fig. 2:**
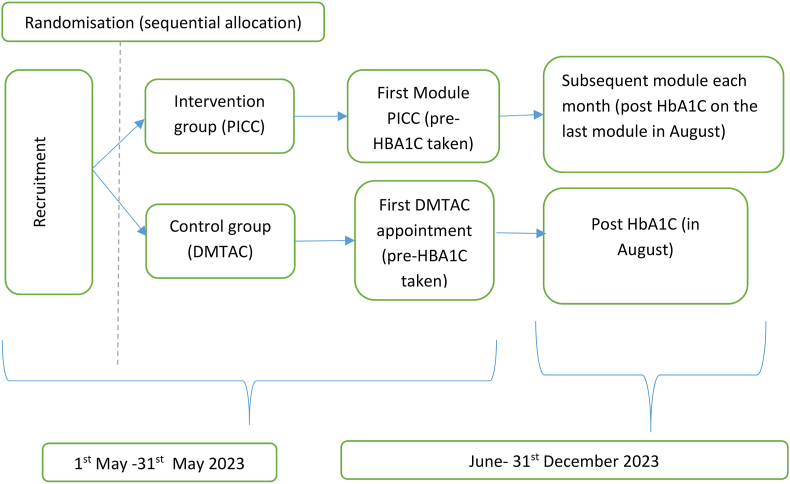
Study timeline.

Participants will undergo screening and recruitment between 1 May and May 31, 2023. The intervention will be implemented across all facilities from 1 May to December 31, 2023. Pre-measurement of HbA1C is scheduled for the initial and final sessions with participants. Refer to Fig. 2 for the study timeline and Fig. 1 for the study procedure. Both the PICC and DMTAC groups will have the MYMAAT and modified DKT assessments administered by facilitators and DMTAC-trained pharmacists during their initial and final sessions.

### 2.15 Outcome measurement

The primary objective of the study is to assess compliance and knowledge related to diabetes medication management using the Malaysia Medication Adherence Assessment Tool (MYMAAT) questionnaire and the Modified Diabetes Knowledge Test (DKT) questionnaire (Malay version). The secondary objective is to evaluate the effectiveness of the primary outcome by measuring changes in HbA1C levels. The instruments used to measure these primary and secondary outcomes are detailed in Table 3.

**Table 3:** Outcome measure of this study.

Variables	Number of items	Measuring Scales
Primary outcome		
Medication adherence (MYMAAT questionnaire)	12	5-point Likert Scales
Diabetes Knowledge test (Modified DKT-Malay version)	20	Three choice answer: Correct, wrong, and Do not know
Secondary outcome		
HBA1C	1	Percentage of glycated haemoglobin of total haemoglobin (laboratory-analysed)

### 2.16 Informed consent/assent process

The informed consent process commences with the distribution of consent forms, wherein the study's objectives are elucidated to participants. They are assured of the voluntary nature of their participation and the option to withdraw without consequences. The information collected is exclusively for research purposes and is restricted to the principal investigators. In case of any modifications, participants will be promptly notified via phone. Facilitators and DMTAC pharmacists initially provided the informed consent forms, clarifying the study's intent to seek permission. Participants are apprised of their voluntary involvement, the right to discontinue or decline, and the confidentiality of information accessible solely by principals and co-investigators.

### 2.17 Statistical analysis

Data analysis will be executed utilising SPSS, focusing on descriptive statistics collected through the CRF. Before the main study, the content validity of the CRF will be meticulously examined, incorporating insights from three expert opinions. Statistical analyses will include the assessment of differences between intervention and control groups at baseline, with specific emphasis on DM knowledge and HbA1c levels. T-tests and chi-square tests will be employed for relevant comparisons. An intention-to-treat analysis will encompass all participants. ANOVA will explore relationships within the data, ensuring a robust and comprehensive analysis approach aligned with the study's objectives.

## 3 Discussion

Assessing the efficacy of the Pharmacy Integrated Community Care (PICC) model is essential to determine its impact on patient health outcomes. This collaborative approach, involving diverse healthcare professionals like pharmacists, optimises diabetes patient care through comprehensive medication management and health monitoring. Evaluating PICC's effectiveness is crucial for reducing healthcare costs, improving patient satisfaction, and guiding its optimal implementation within broader healthcare systems.

A pivotal research facet is selecting theoretical frameworks to underpin the model construction. In alignment with previous studies in the field, we have adopted the health belief model and the social cognitive theory as the foundational frameworks for our model [23]. The health belief model delves into individuals' perceptions and attitudes toward their health and their appraisal of health threats, influencing their health-related behaviours [[33], [34], [35], [36], [37]]. Conversely, the social cognitive theory underscores the interplay between individual behaviours, environmental factors, and personal attributes that shape behaviours [37,38].

The amalgamation of the health belief model and the social cognitive theory within our model aims to comprehensively comprehend the factors steering health behaviours pertinent to chronic disease prevention and management [37]. Through the health belief model, we dissect how individuals perceive their susceptibility to chronic diseases and the determinants that mould their convictions about the efficacy of preventive behaviours [[33], [34], [35], [36], [37]]. Concurrently, the social cognitive theory illuminates the intricate interplay between individual behaviours, surroundings, and personal attributes that impact individuals' capacity to adopt healthful behaviours [37,38]. This integration furnishes our model with a valuable framework for devising interventions that foster salutary behaviours and deter chronic ailments [37].

The current study employs randomised trials to gauge the efficacy of the PICC intervention, juxtaposed with Diabetes Medication Therapy Adherence Counseling (DMTAC), the gold standard and control. This methodological choice facilitates the fusion of qualitative and quantitative data, which is pivotal for a comprehensive grasp of the phenomenon. The holistic approach adopted in this study proffers a robust and nuanced framework for delving into the intricate dynamics at play. Moreover, this approach harbours potential adaptability to diverse settings and contexts in future research, cementing its applicability and relevance.

The proposed study boasts several strengths. Firstly, it promises pivotal insights into the efficacy of the PICC program in heightening diabetes management and ameliorating HbA1C levels. Secondly, the study design encompasses a stringent randomised controlled trial setup, enabling causal inferences. The study assesses various outcomes, including diabetes knowledge and medication adherence. Fourthly, the study serves as a beacon to elucidate the theoretical frameworks that underpin group-based diabetes interventions.

### 3.1 Limitations

Nevertheless, it is crucial to acknowledge certain limitations inherent in our study design. Firstly, there is a potential for selection bias due to excluding patients who may have been disinclined or unable to participate in a randomised controlled trial. Additionally, since the administration of the Case Report Form (CRF) was conducted by trained facilitators who were part of the study team, there is a possibility of bias during the assessment of MyMAAT and Modified DKT. While efforts were made to mitigate bias through training and standardisation, this aspect warrants consideration.

Furthermore, the findings of our study may be limited in their generalizability to broader populations beyond our study sample owing to our participants' specific characteristics and circumstances. Although randomised controlled trials are considered the gold standard for intervention evaluation, our study's relatively short follow-up period may restrict the assessment of long-term outcomes.

Despite our attempts to minimise bias by blinding participants and assessors to group assignment, the potential influence of unmeasured confounding variables on the results cannot be entirely ruled out. Future studies could benefit from additional measures to address these limitations and enhance the robustness of the findings.

## 4 Conclusion

In summation, this study wields the potential to exert substantial influence on managing chronic illness patients, particularly those necessitating prolonged intravenous therapy. Evaluating the efficacy of Pharmacy Integrated Community Care (PICC) can generate evidence-based recommendations that can reshape patient outcomes and alleviate healthcare expenses. The insights from this study might well impact clinical practice guidelines in chronic illness management, ushering in consequential implications for patient care. The study's findings could also aid healthcare systems in judicious resource allocation and determining optimal care models for patients with intricate healthcare requisites. Ultimately, the ramifications of this study extend beyond the specific patient cohort under scrutiny, offering advantages to a broader spectrum of individuals grappling with chronic illnesses that necessitate comprehensive healthcare management.

## Disclaimers

The information and findings presented in this journal article are based on the research conducted by the authors and are intended for informational and scholarly purposes only. The views, opinions, and conclusions expressed in this article are those of the authors and do not necessarily reflect the views of the journal, its editorial board, or the institutions with which the authors are affiliated. The information and findings presented in this journal article are based on the research conducted by the authors and are intended for informational and scholarly purposes only. The views, opinions, and conclusions expressed in this article are those of the authors and do not necessarily reflect the views of the journal, its editorial board, or the institutions with which the authors are affiliated.

## Source(s) of support

This research received no specific grant from funding agencies in the public, commercial, or not-for-profit sectors.

## Disclosure of relationships and activities

I declare that all the information in the ICMJE Disclosure Form is accurate and complete. I have disclosed all relevant relationships and activities, both financial and non-financial, as per the guidelines set forth by the International Committee of Medical Journal Editors (ICMJE). This declaration affirms my commitment to transparency and ethical reporting of potential conflicts of interest in this manuscript.

I hereby declare that I have no financial or personal interests that could be perceived as potential conflicts of interest, and I hold no beliefs that may influence the objectivity of this work. Should there be any uncertainty, I am ready to provide further clarification to ensure complete transparency. I understand the importance of accurate and open communication and am committed to adhering to the highest ethical standards throughout the publication.

I hereby declare that the submission of this article signifies that the research work described within has not been previously published, except in the form of an abstract, a published lecture, or an academic thesis, as outlined in the guidelines regarding multiple, redundant, or concurrent publications. Furthermore, I affirm that this manuscript is not under consideration for publication elsewhere and that all authors have given their approval for its submission. This declaration is made with the understanding that, if accepted, the manuscript will not be published elsewhere in the same form, either in English or any other language, including electronic formats, without obtaining the written consent of the copyright holder.

## Publication policy

Participants' privacy remains paramount, with no personal details disclosed in study publications to ensure anonymity.

## Registration

The trial, registered as NMRR-21-767-59240, adheres to ethical standards and is overseen by Malaysia's Medical Research and Ethics Committee (MREC).

## Protocol

Access Protocol NCT05106231 on ClinicalTrials.gov for comprehensive study details, fostering transparency and collaboration among researchers and the public.

## Funding

This research was conducted without specific grants and utilised existing resources from the PICC Program without additional 10.13039/100009647Ministry of Health funding.

## CRediT authorship contribution statement

**Kamarudin Ahmad:** Writing – review & editing, Writing – original draft, Visualization, Resources, Project administration, Methodology, Investigation, Formal analysis, Data curation, Conceptualization. **Lawrence Anchah:** Writing – review & editing, Validation, Supervision, Methodology, Conceptualization. **Chuo Yew Ting:** Writing – review & editing, Supervision, Methodology, Conceptualization. **Su Ee Lim:** Resources, Project administration, Investigation, Data curation.

## Declaration of competing interest

The authors declare that they have no known competing financial interests or personal relationships that could have appeared to influence the work reported in this paper.

## Data Availability

No data was used for the research described in the article.
