# Supplementary material for: Effectiveness of a pharmacist-led, community group-based education programme in enhancing diabetes management: A multicentre randomised control trial
Source: Contemp Clin Trials Commun. 2024 Feb 24;38:101280. doi: 10.1016/j.conctc.2024.101280 (PMC10909608; doi:10.1016/j.conctc.2024.101280)
Supplement: Multimedia component 1 [file mmc1.docx]

**Appendix 1: Contents of structured PICC programme**

| **MODUL1: INTRODUCTION TO DIABETES**  Number of participants: 10 people (minimum)  Objective:  1. Create a support group that helps increase patients’ self-motivation in controlling diabetes  2. Recognize and understand diabetes  3. Share the information learned among the participants | | | | | | | | |
| --- | --- | --- | --- | --- | --- | --- | --- | --- |
| **Duration** | **Objective** | | **Activity** | | **Person in-charge** | | **Materials** | **Procedure** |
| 10 minutes | Registration | | - | | - | | - Consent form - Patient - Patient’s Information record PICC - myPICC Activity Book | - Write the name on the card (name card) - Distribute consent forms (2 copies) to participants, one copy PF keep, one copy participants, keep - Distribute “PICC Participant Information Record” & “MyPICC Activity Book” to participants - Ask them to fill in demographic data in both books |
| 5 minutes | Briefing PICC | | Lecture | | Head facilitator | | - | - Information about PICC (objective, etc.) |
| 30 minutes | Clinical impact monitoring | | Health screening | | Medical team | | - Health screening kit - Outsource medical team (MA / Nurse / etc.) to help do the health screening - Set up counters to do the health screening | - Explain to participants why we need to do a health screening, what are the things or parameters we are going to measure - The medical team will conduct the following health screenings: - Body Mass Index (BMI) - Blood sugar levels (fasting blood sugar and HbA1c) - Blood pressure level |
| 15 minutes | Ice-breaking | | Group activity | | Head facilitator | | - Cards for name tags | - Participants are divided into groups (maximum five groups) - Each group will be given a card for participants to write their names. - The facilitator will start the ice-breaking session by asking questions:  1. Name 2. Origin 3. Who do you live with (alone / family or with non-family) 4. o History of diabetes |
| 25 minutes | Participants’ understanding of diabetes | | Brainstorming & Clustering | | Facilitator | | - Card - Pen marker - Large paper (if any) - Sellotape | - The facilitator gives out one card and one pen to 2 participants (2 participants share one card and one pen, discuss) - The facilitator asks some questions:   o Participants’ perceptions of diabetes  o Factors/causes of getting diabetes  o Expectations or plans of participants  when joining PICC   - Participants’ answers are recorded on the card. - Give participants 1 minute to write down the answer/idea on the card - After that, each facilitator arranges the ideas (clustering) by category/problem on Large paper or whiteboard or wall with stickers (sellotape) - The facilitator rectifies the “expectations or plans.” - Perceptions, factors, rectify later after the module 1 lecture |
| 20 minutes | Module 1: Introduction to Diabetes | | Lecture | | Facilitator | | - Module 1 Slides: Introduction to Diabetes - myPICC Activity Book - Other teaching aids | - Briefing and discussion sessions conducted by the facilitator using Module 1: Introduction to Diabetes (slides or checklists) - The facilitator guides the participants to complete the activities for Module 1 in the myPICC Activity Book. - Assist the participants in doing the activities in the MyPICC Activity Book after almost every slide. (page 4 – 7) - The facilitator should also comment on the participants ’understanding of diabetes based on the ideas on the cards that have been clustered. - Representatives of participants from each group may be asked to provide additional comments. |
| 10 minutes | Module 1: Introduction to Diabetes | | Group work | | Facilitator | | - Module 1 Slides: Introduction to Diabetes - myPICC Activity Book | - The facilitator arranges the appropriate place as:  1. Blood vessels (road) 2. Cell (home)  - The facilitator identifies the role of the participant in the role-play session:  1. Glucose (passenger) – 8 participants 2. Insulin (car) – 2 participants  - Perform role-play activities for two situations: normal individuals & diabetics. |
| 5 minutes | Conclusion for session 1 | | Lecture | | Facilitator | | - Module 1 Slides: Introduction to Diabetes - myPICC Activity Book | - Record the date of session 2 in myPICC Activity Book - The facilitator reminded the participants to bring their medicine in session 2. |
| 5 minutes | Participant comprehension test | | Pop quiz | | Facilitator | | - myPICC Activity Book | - Each participant is asked to complete a quiz question (page 8) included in the myPICC Activity Book - The facilitator will check the answers given. - Scores obtained will be recorded on page 2 MyPICC Activity Book & Page 2 Participant Information Record book. |
| **MODULE 2: TAKING ANTIDIABETES MEDICINES**  Number of participants: 10 people (minimum)  Target group: PICC participants  Objective:  1. Recognize and understand the indications of DM Medication  2. Explain the concept of 5B in taking DM medication  3. Share the information learned among the participants | | | | | | | | |
| **Duration** | | **Objective** | **Activity** | | | **Person in-charge** | **Materials** | **Procedure** |
| 30 minutes | | Clinical impact monitoring | Health screening | | | Medical team | - Health screening kit - Outsource the medical team (MA / Nurse / etc.) to help do the health screening - Set up counters to do the health screening | - The medical team will conduct the following health screenings:  1. Body Mass Index (BMI) 2. Fasting blood sugar (fasting blood sugar) 3. Blood pressure level |
| 15 minutes | | Recap for session 1 | Generate ideas | | | Facilitator | - Cards & Pens | - The facilitator obtains feedback from participants on session 1. - Optional: Ask participants/patients to save the book (cannot refer to the book for answers) - 2 – 3 participants (patients) are given 1 card & 1 pen - After that, the facilitator asks questions:  1. How does diabetes occur? 2. What causes diabetes? 3. What is the target sugar level for diabetics?  - Patients are given 1 minute to write the answer on the card. - Cards will be collected and clustered on a Large paper/wall/whiteboard. - The facilitator will comment based on the participants’ answers. |
| 10 minutes | | Participants’ understanding of diabetes medication intake | Brainstorming | | | Facilitator | - Card - Pen marker - Mahjong paper (if any) - Sellotape | - The facilitator gives out one card and one pen to 2 participants (2 participants share one card and one pen, discuss) - The facilitator asks some questions:  1. How do you take diabetes medication? (before, during or after meals) 2. Where do you store diabetes medicine?  - Participants’ answers are recorded on the card. - Give participants 1 minute to write down the answer/idea on the card - After that, each facilitator arranges ideas (clustering) according to categories/answers on Large paper or whiteboard or wall with stickers (sellotape) - Rectify later after the module 2 talks |
| 30 minutes | | Module 2: Antidiabetic Medication Management and conclusions for session 2 | Lecture | | | Facilitator | - Module 2 Slide: Taking Antidiabetic Medications - myPICC Activity Book - Other teaching aids | - Briefing and discussion sessions conducted by the facilitator using Module 2: Taking Diabetes Medication (slides or checklists) - The facilitator guides the participants to complete the activities for Module 2 in the myPICC Activity Book. - Assist the participants in doing the activities in the MyPICC Activity Book after almost every slide. (pages 10 – 15) - The facilitator should also comment on the participants’ understanding of taking diabetes medication based on the ideas on the cards that have been clustered. - Representatives of participants from each group may be asked to provide additional comments. - Optional: Brainstorming & clustering for factors causing hyperglycemia and hypoglycemia can be done. - Dates for session three will be stated in the myPICC Activity Book. |
| 5 minutes | | Participant comprehension test | Pop quiz | | | Facilitator | - myPICC Activity Book | - Each participant is asked to complete the quiz (page 16) included in the myPICC Activity Book - The facilitator will check the answers given. - Scores obtained will be recorded on page 2 MyPICC Activity Book & Page 2 Participant Information Record book. |
| **MODULE 3: COMPLICATIONS OF DIABETES**  Number of participants: 10 people (minimum)  Target group: PICC participants  Objective:  1. Recognize and understand the complications of diabetes  2. How to prevent and control the complications of diabetes  3. Share the information learned among the participants | | | | | | | | |
| **Duration** | | **Objective** | **Activity** | | | **Person in-charge** | **Materials** | **Procedure** |
| 30 minutes | | Clinical impact monitoring | Health screening | | | Medical team | - Health screening kit - Outsource the medical team (MA / Nurse / etc.) to help do the health screening - Set up counters to do the health screening | - The medical team will conduct the following health screenings:  1. Body Mass Index (BMI) 2. Fasting blood sugar (fasting blood sugar) 3. Blood pressure level |
| 15 minutes | | Recap for session 2 | Generate ideas | | | Facilitator | - Card | - The facilitator gets feedback from the participants about session 2. - Optional: Ask participants/patients to save the book (cannot refer to the book for answers) - 2 – 3 participants (patients) are given 1 card & 1 pen - The facilitator asks questions:  1. Types of diabetes medications 2. Time of insulin intake 3. Symptoms of hypoglycemia and hyperglycemia  - Patients are given 1 minute to write the answer on the card. - Cards will be collected and clustered on a Large paper/wall/whiteboard. - The facilitator will comment based on the participants’ answers. |
| 10 minutes | | Participants’ understanding of the complications of diabetes | Brainstorming | | | Facilitator | - Card - Pen marker - Mahjong paper (if any) - Sellotape | - The facilitator gives out one card and one pen to 2 participants (2 participants share one card and one pen, discuss) - The facilitator asks some questions:  1. Why do complications of diabetes occur? 2. What are the common complications of diabetes you see in people with diabetes?  - Participants’ answers are recorded on the card. - Give participants 1 minute to write down the answers/ideas on the card - After that, each Facilitator arranges ideas (clustering) by category/answer on Large paper or whiteboard or wall with stickers (sellotape) - Rectify later after the module 3 Lecture |
| 30 minutes | | Module 3: Diabetes Complications: Description and conclusions for session 3 | Lecture | | | Ketua Sesi / Facilitator | - Module 3 Slide: Complications of Diabetes - myPICC Activity Book - Other teaching aids | - Briefing and discussion sessions conducted by the facilitator with the help of Module 3: Diabetes Complications (slides or checklists) - The facilitator guides the participants to complete the activities for Module 3 in the myPICC Activity Book. - Assist the participants in doing the activities in the MyPICC Activity Book after almost every slide. (pages 18 – 20) - Emphasise the “information” on foot care on page 20 in the MyPICC Activity Book. (not in the slide) - The facilitator should also comment on the participants’ understanding of the complications of diabetes based on the ideas on the cards that have been clustered. - Representatives of participants from each group may be asked to provide additional comments. - The date for session 4 will be stated in the myPICC Activity Book. |
| 5 minutes | | Participant comprehension test | Pop quiz | | | Facilitator | - myPICC Activity Book | - Each participant is asked to complete a quiz question (page 21) included in the myPICC Activity Book. - The facilitator will check the answers given. - Scores obtained will be recorded on page 2 MyPICC Activity Book & Page 2 Participant Information Record book. |
| **MODULE 4: PRACTICE A HEALTHY LIFESTYLE**  Number of participants: 10 people (minimum)  Target group: PICC participants  Objective:  1. Provide exposure to the diet and healthy lifestyle of diabetics.  2. Share the information learned among the participants. | | | | | | | | |
| **Duration** | **Objective** | | **Activity** | **Person in-charge** | | | **Materials** | **Procedure** |
| 30 minutes | Clinical impact monitoring | | Health screening | Medical team | | | - Health screening kit - Outsource the medical team (MA / Nurse / etc.) to help do the health screening - Set up counters to do the health screening | - The medical team will conduct the following health screenings:  1. Body Mass Index (BMI) 2. Fasting blood sugar (HbA1c) 3. Blood pressure level |
| 20 minutes | Morning exercise | | Aerobic | Physiotherapist | | | - Music | - Each participant will participate in an exercise led by an aerobics instructor. |
| 5 minutes | Recap for session 3 | | Generate ideas | Facilitator | | | - Card | - The facilitator gets feedback from the participants about session 3. - 2 – 3 participants (patients) are given 1 card & 1 pen - The facilitator asks questions:  1. How to reduce the risk of getting diabetes complications?  - Patients are given 1 minute to write the answer on the card. - Cards will be collected and clustered on a Large paper/wall/whiteboard. - The facilitator will comment based on the participants’ answers. |
| 10 minutes | Healthy lifestyle | | Lecture  (Slide no. 36 – 40) | Physiotherapist | | | - Module 4 Slides: - Practice healthy lifestyle - myPICC Activity Book | - Briefing and discussion sessions conducted by physiotherapists using Module 4: Practicing a Healthy Lifestyle (slides or checklists) - Participants complete the activities for Module 4 in the myPICC Activity Book. (pages 23 – 24) |
| 35 minutes | Diet for diabetics | | Lecture  (Slide no. 41 – 45) | Dietician | | | - Module 4 Slides: Practicing a Healthy Lifestyle - Teaching aids (example: bowl, plate or spoon to measure the quantity of food) - myPICC Activity Book | - Information sessions and discussions conducted by nutrition science officers using Module 4: Practicing a Healthy Lifestyle (slides or checklists) - The facilitator guides the participants to complete the activities for Module 4 in the myPICC Activity Book. (page 24 – 26) - Demonstrations are shown to reinforce participants ’understanding. |
| 15 minutes | PICC summary | | Generate ideas | Facilitator | | | - Card & pen - Large paper - Adhesive tape | - Participants will be divided into small groups. - Each group will be given 3 pieces of cards, and the answer is written on the card. - The facilitator will ask questions:  1. What causes diabetes? 2. How to control diabetes? 3. What is the targeted sugar level for diabetics?  - Cards will be collected, and the facilitator will do clustering. - The facilitator will comment on the answers from the participants. - The facilitator will make a summary of the PICC and the performance of the participants throughout participating in the PICC. |
| 5 minutes | Participant comprehension test | | Pop quiz | Facilitator | | | - myPICC Activity Book | - Each participant is asked to complete the quiz questions included in myPICC Activity Book (page 27) - The answers given will be checked by the facilitator. - The marks obtained will be recorded in the PICC Participant Information Record and MyPICC Activity Book. |
| 5 minutes | PICC Program Feedback | | Customer satisfaction survey | Facilitator | | | - Google Form | - Each participant is asked to complete a customer satisfaction survey as in the link. (page 28 MyPICC Activity Book) |
| 10 minutes | Certificate of Participation Presentation Session | | - | - | | | - Certificate | - Certificate of participation is given to all participants who successfully attend 3 of the 4 sessions of the PICC program. |
