# Supplementary material for: Effectiveness of a pharmacist-led, community group-based education programme in enhancing diabetes management: A multicentre randomised control trial
Source: Contemp Clin Trials Commun. 2024 Feb 24;38:101280. doi: 10.1016/j.conctc.2024.101280 (PMC10909608; doi:10.1016/j.conctc.2024.101280)
Supplement: Multimedia component 2 [file mmc2.docx]

**MOH/S/FAR/71.22(GU)-e**

Appendix 2: Diabetes Medication Therapy Adherence Clinic Protocol


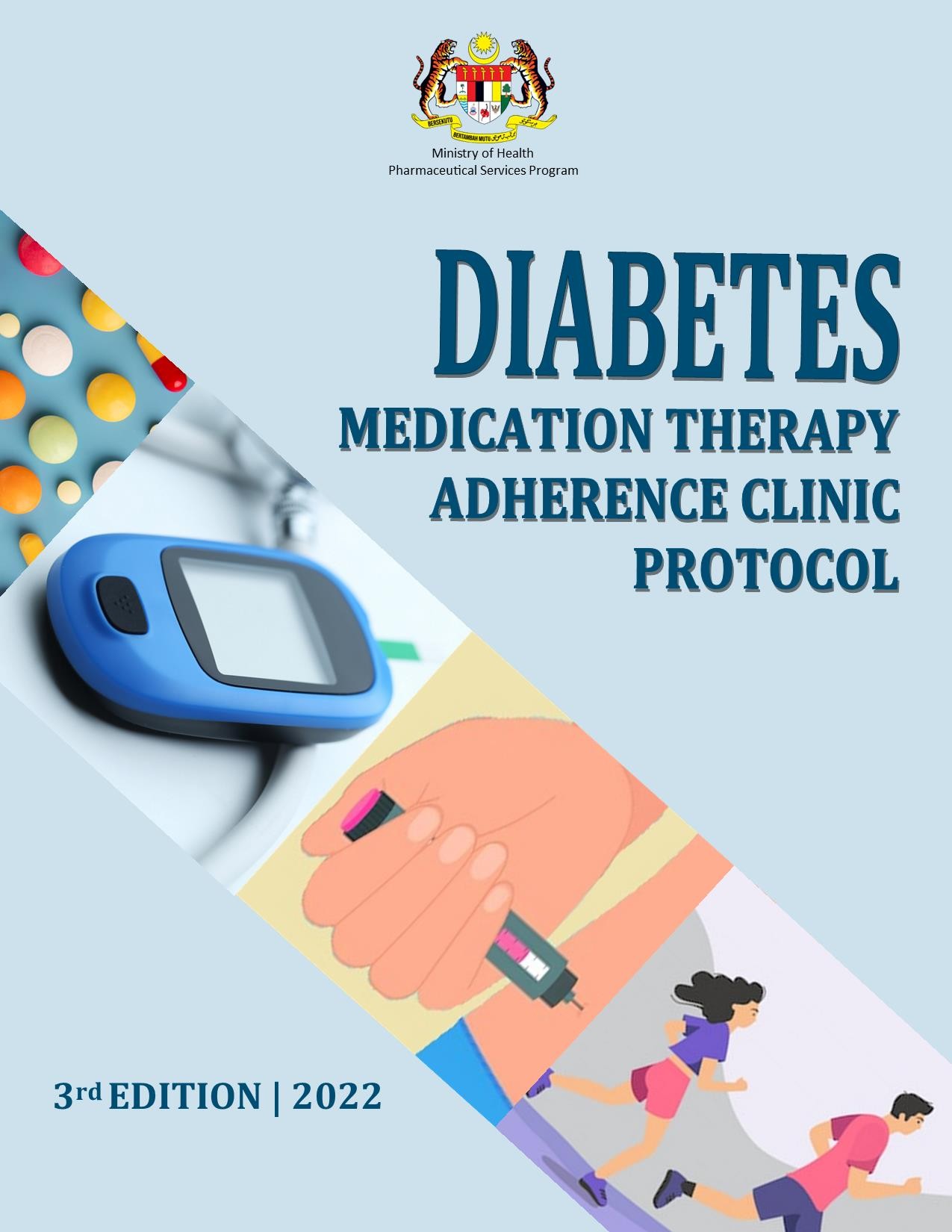

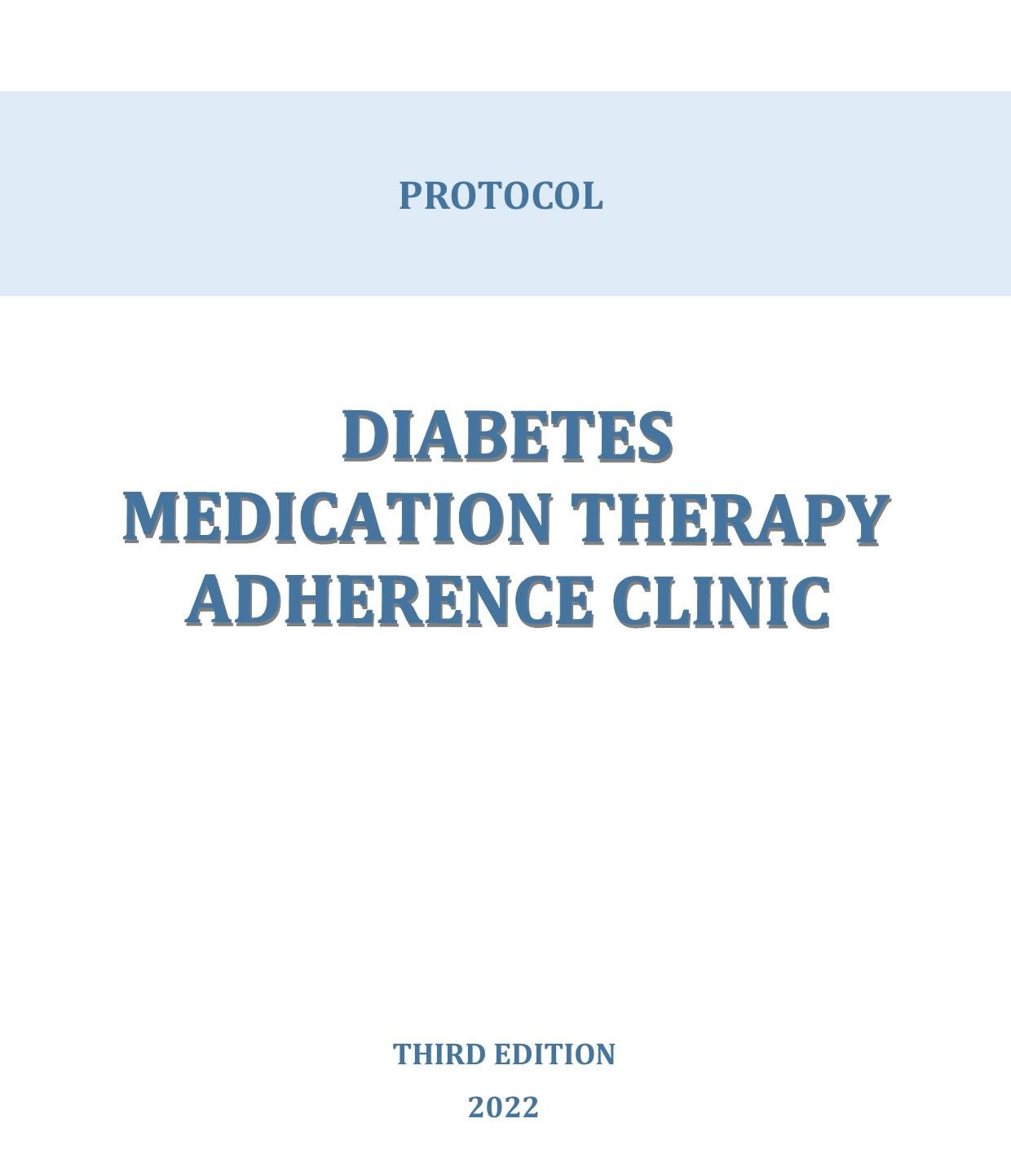


**Third Edition 2022**

**December 2022**

**Published by: Pharmaceutical Services Program**

**Ministry of Health, Malaysia**

Lot 36, Jalan Profesor Diraja Ungku Aziz,

46200 Petaling Jaya, Selangor, Malaysia

Tel: 603-7841 3200

Website: [www.pharmacy.gov.my](http://www.pharmacy.gov.my)

**© ALL RIGHTS RESERVED**

No part of this publication may be reproduced, stored, or transmitted in any form or by any means whether electronic, mechanical, photocopying, tape recording, or others without prior written permission from the Senior Director of Pharmaceutical Services Program, Ministry of Health, Malaysia.


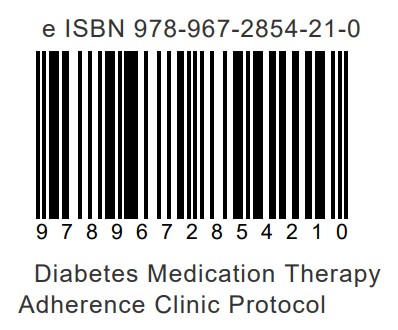


**FOREWORD**

**FUZIAH BINTI ABDUL RASHID**

Director

Pharmacy Practice & Development Division

Ministry of Health Malaysia


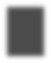

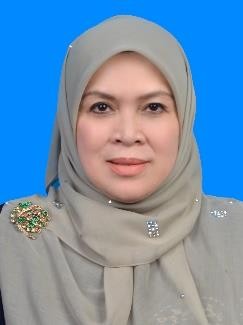


According to National Health and Morbidity Survey (NHMS) 2019, 1 in 5 adults in Malaysia which account for about 3.9 million people aged 18 years and above suffer from diabetes. The prevalence rate of diabetes in adults has increased from 13.4% in 2015 to 18.3% in 2019 and this is a worrying trend.

Pharmacists in diabetes care teams have been shown to play a vital role in diabetes management by not only improving patient outcomes but can also help prevent complications. With the ever-increasing treatment options for diabetes over the past few years, pharmacists are best positioned to help patients understand the fundamentals and complexities regarding the medication as well as help them understand how to manage their condition better. This is mirrored in the latest edition of the Diabetes Medication Therapy Adherence Clinic (DMTAC) Protocol.

This third edition of the DMTAC Protocol is the product and hard work of the Clinical Pharmacy Working Committee (Diabetes Mellitus Subspecialty), and I would like to applaud all of the members for their effort. This protocol can hopefully serve as a guideline to all pharmacists involved in DMTAC activities.

Thank you.

**ADVISOR**

**Fuziah Bt Abdul Rashid**

*Director*

*Pharmacy Practice and Development Division*

*Pharmaceutical Services Program, MOH*


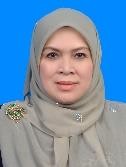


**EDITORS**

**Rohana Bt Hassan**

*Deputy Director Clinical and Technical Pharmacy Pharmacy Practice and Development Division Pharmaceutical Services Program, MOH*


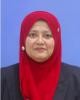


**Masfiza Bt Abdul Hamid**

*Senior Principal Assistant Director*

*Pharmacy Practice and Development Division*

*Pharmaceutical Services Program, MOH*


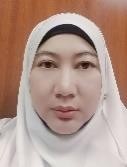


**Siti 'Aqilah Bt Mohd Nordin**

*Principal Assistant Director*

*Pharmacy Practice and Development Division*

*Pharmaceutical Services Program, MOH*


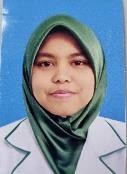


**EXTERNAL REVIEWERS**

**Datuk Dr Zanariah Hussein** *Head of Endocrinology Services Ministry of Health Malaysia*


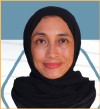


**Dr Florence Tan Hui Sieng**

*Consultant Endocrinologist & Physician*

*Sarawak General Hospital*


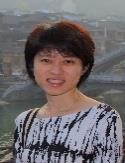


**Dr Wan Fadhilah Binti Wan Ismail** *Family Medicine Specialist Mahmoodiah Health Clinic*


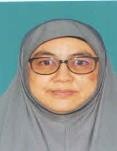


**Dr Noraida Binti Abdul Rahman**

*Family Medicine Specialist*

*Kuala Pilah Health Clinic*


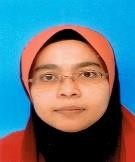


**Afizah Bt Arsad**


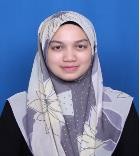

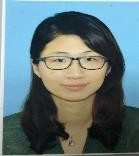

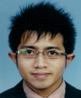

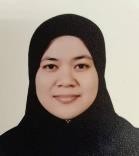

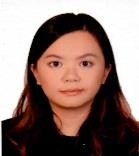

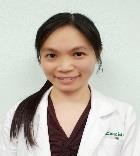

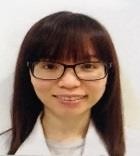

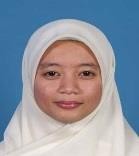


*Hulu Langat Public Health Office, Selangor*


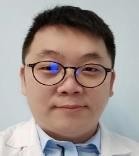


**Annie Soong Tse Yeen** *Duchess of Kent Hospital, Sabah*

**Brendy Lee Wai Han**

*Tuanku Ampuan Najihah Hospital, Negeri Sembilan*


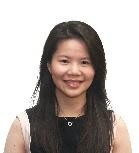


**Fiona Tong Hui Ling** *Kuala Lumpur Hospital, Kuala Lumpur*

**Khairunnisa Bt Zamri** *Sultanah Nur Zahirah Hospital, Terengganu*

**Mohd ‘Izzat Bin Ismorning** *Sultanah Bahiyah Hospital Kedah*

**Ng Sin Ye**

*Tengku Ampuan Afzan Hospital, Pahang*

**Noorul Aimi Bt Daud** *Serdang Hospital, Selangor*


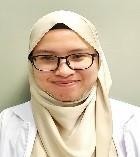


**Albert Ting Siong Hung** *Petra Jaya Health Clinic, Sarawak*

**Aziani Bt Yacob**

*Raja Perempuan Zainab II Hospital*

*Kelantan*

**Cheryl Yong Wai Yin**


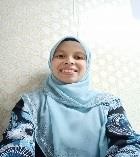


*Raja Permaisuri Bainun Hospital, Perak*

**Go Kai Ying** *Kulim Hospital, Kedah*

**Lim Phei Ching**


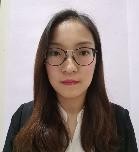


*Pulau Pinang Hospital, Pulau Pinang*

**Dr Navin Kumar Loganadan**


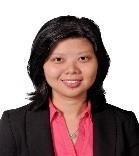


*Putrajaya Hospital,*

*Federal Territory of Putrajaya*

**Noor Rodhiah Bt Abd Rahman** *Sultanah Nur Zahirah Hospital, Terengganu*


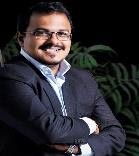


**Nur Diniah Bt Shaharum**


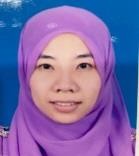


*Melaka Hospital, Melaka*

**Seow Cui Jiun**

*Sultanah Bahiyah Hospital*

*Kedah*

**Tai Chia Woon** *Mahmoodiah Health Clinic, Johor*


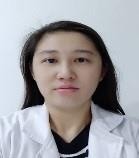


**Dr Shamala Ayadurai** *Sultanah Aminah Hospital, Johor*

**Tan Huay Synn**


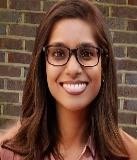


*Tengku Ampuan Rahimah Hospital,*

*Selangor*


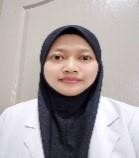

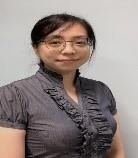

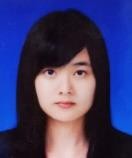


**Wan Ruwaida Bt Wan Mokhtar**

*Tuanku Fauziah Hospital, Perlis*

**TABLE OF CONTENT**

| **A.** | Introduction | 1 |
| --- | --- | --- |
| **B.** | Objectives | 3 |
| **C.** | Scope of Service | 3 |
| **D.** | Manpower Requirement | 3 |
| **E.** | Appointment | 3 |
| **F.** | Outcome Measurement | 4 |
| **G.** | Workflow | 4 |
|  | 1. Patient Selection | 4 |
|  | 2. Initial Assessment by the DMTAC Pharmacist | 6 |
|  | 3. Second and Subsequent Visits | 7 |
|  | 4. DMTAC Patients Registry | 8 |
|  | 5. Missed Visit | 8 |
|  | 6. Pharmaceutical Review | 8 |
|  | 7. Medication Dispensing And Counselling | 9 |
|  | 8. Documentation | 9 |
|  | 9. Discharge Criteria | 10 |
| **H.** | References | 11 |
| **I.** | Appendices | 12 |
|  | 1. Diabetes MTAC Workflow (First visit) | 13 |
|  | 2. Diabetes MTAC Workflow (Subsequent visit) | 14 |
|  | 3. Diabetes MTAC Workflow (Phone/Virtual call) | 15 |
|  | 4. Education Modules for Diabetes Patients | 16 |
|  | 5. Malaysia Medication Adherence Assessment Tool | 17 |
|  | 6. Pharmacy Information System (PhIS) Documentation | 20 |
|  | 7. DMTAC Pharmacotherapy Review Form | 30 |
|  | 8. DMTAC Session Checklists | 35 |
|  | 9. Abbreviations | 36 |

**A. INTRODUCTION**

The International Diabetes Federation reported that there are 537 million people with diabetes around the world and this prevalence is likely to increase steadily resulting in diabetes being a major healthcare burden for most countries (International Diabetes Federation, 2021). According to the National Health and Morbidity Survey (2019) conducted by the Ministry of Health Malaysia, 3.9 million Malaysians aged 18 years old and above or one in five (18.3%) adults in Malaysia has Type 2 Diabetes (T2D). This alarming finding urges us to manage and educate patients with DM aggressively to prevent further increases trends in DM prevalence.

The findings from the United Kingdom Prospective Diabetes Study (UKPDS) showed that with every 1% reduction in HbA1c, there was a 21% reduction in death related to diabetes, a 14% reduction in the incidence of myocardial infarction, and a 37% reduction in the incidence of microvascular complications (Stratton et al,

2000). Optimal benefits from medications can only be achieved through good adherence which is a vital component of diabetes management. Poor medication adherence has been associated with increased cases of uncontrolled diabetes (Hammad et al, 2017). There is also evidence for improved glycemic control indicated by reduced HbA1c levels with increased medication adherence (Rozenfeld et al, 2008).


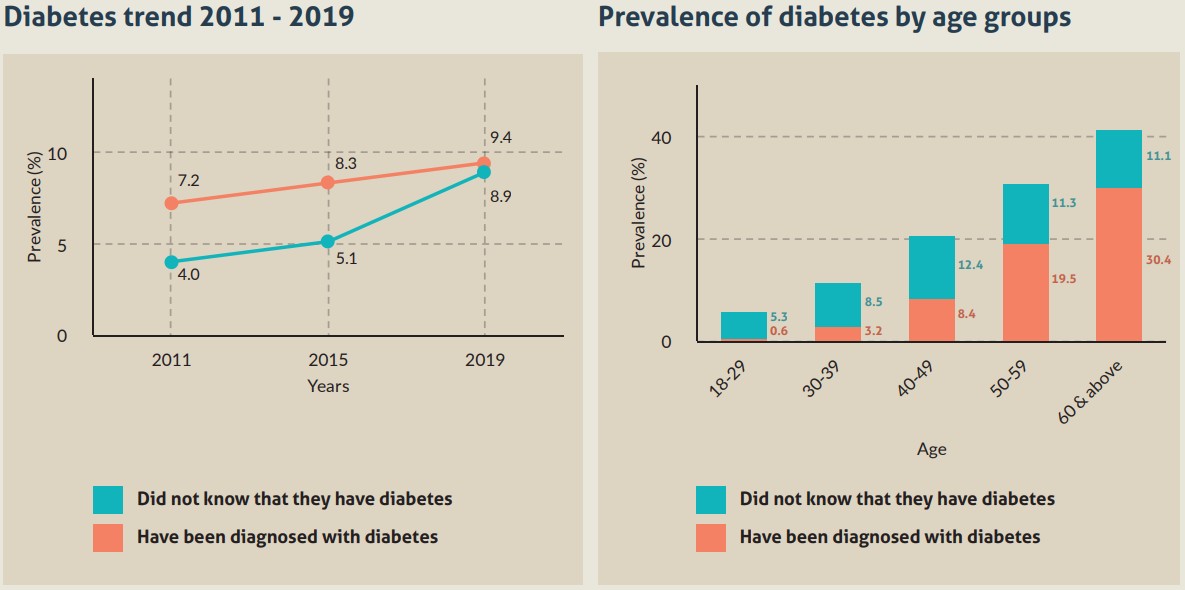


Figure 1: Prevalence of Type 2 Diabetes in Malaysia (NHMS 2019)

Diabetes Medication Therapy Adherence Clinic (DMTAC) is an ambulatory care service offered by pharmacists in collaboration with physicians to help patients with DM to achieve medication adherence and obtain good glycaemic control. Patients enrolled will be followed up for a minimum of four visits where they will receive medication adherence assessment, identification, and management of drug-related problems, medication counselling, monitoring of clinical outcomes, and diabetes education by the pharmacist.

The outcomes of pharmacist-led diabetes clinics have reported significant reductions in HbA1c, which is an outcome measure of glycaemic control. A

randomised clinical trial involving 217 Type 2 diabetes patients who received

DMTAC PROTOCOL 3rd EDITION 2022 1


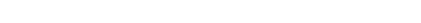

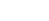

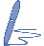

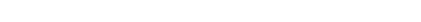

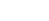

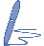


pharmacist care for 12 months in the United States showed a 2.5% significant reduction in HbA1c (Rothman et al, 2005). In Australia, the Pharmacy Diabetes Care Program run by community pharmacists have shown improvements in glycemic control of diabetes patients with HbA1c reductions of 0.9% after 6 months of being in the program. A similar study in Northern Cyprus also showed a significant 0.74% reduction in HbA1c after a 12 months follow-up with the pharmacists (Korcegez et al, 2017).

The impact of DMTAC run by pharmacists in Ministry of Health in Malaysian type

2 diabetes patients have been reported through several local studies. A study conducted in Hospital Pulau Pinang demonstrated that DMTAC improved glycaemic control by 1.73% reduction in HbA1c and 2.65 mmol/l reduction in fasting blood glucose significantly, reduction in LDL-cholesterol as well as improvement in medication adherence (p<0.05) (Lim et al, 2010). Another prospective randomised open-labelled study conducted in Hospital Pulau Pinang in the year of 2009 to 2010 among 120 diabetes patients also showed that pharmacists managed DMTAC had significantly improved glycaemic control as compared to standard care (Lim et al,

2016). A prospective cohort study in Hospital Kuala Lumpur reported that diabetes patients who underwent DMTAC had significantly greater HbA1c reduction (1.7%) than those who received Standard Care (0.6%) as a result of improvements in medication adherence (p<0.05). The same study also found DMTAC service to be more cost-effective (average cost effectiveness ratio of RM446.01 per 1% HbA1c reduction) than Standard Care (average cost effectiveness ratio of RM1,328.52 per

1% HbA1c reduction) (Loganadan et al. 2012). A multicentre study of patients from Hospital Putrajaya, Hospital Kuala Lumpur, Hospital Pulau Pinang, Hospital Ipoh, Hospital Selayang, Hospital Serdang, Hospital Sultanah Nur Zahirah Kuala Terengganu, Hospital Umum Sarawak and Hospital Queen Elizabeth showed that their HbA1c reduced by 1.0% after 6 months of DMTAC enrolment (p<0.05) (Mohamad et al 2010).

Improved outcome of type 2 diabetes patients enrolled in DMTAC has also been shown in primary care clinics in Ministry of Health. A primary care clinic in Johor reported that DMTAC improved the understanding score of patients towards their medication regimen and showed significant reduction in HbA1c by 1.0% (p<0.05) (Tai, 2016). Furthermore, in a randomized controlled study conducted at a clinic in Sarawak also showed significant improvement in glycaemic control compared to control (-1.58% vs. -0.48%, p<0.05) (Alison et al, 2020). In another study involving

14 health clinics in Kuala Lumpur and Putrajaya, type 2 diabetes patients who attended DMTAC demonstrated a significant 1.0% reduction in HbA1c from 10.7% to 9.7% (You et al, 2015). These evidences lend credence to the fact that pharmacists through the DMTAC clinic play an important role in ensuring medication knowledge and adherence of diabetes patients which results in improved glycemic control. By actively involved in diabetes management, the pharmacists can help target glucose attainment and prevent the development and progression of diabetes related complications. These interventions have also been

proven to be cost-effective to the Ministry of Health.

2 DMTAC PROTOCOL 3rd EDITION 2022

**B. OBJECTIVES**

1. To improve patient’s knowledge of medications and diseases.

2. To increase patient’s adherence to medications.

3. To reduce adverse effects and complications resulting from multiple treatment regimens.

4. To educate patient about diabetes and its complications, proper self- management, medication usage, and self-care devices.

5. To monitor patient’s pharmacotherapy, particularly responses to therapy in between doctor’s visits.

6. To identify pharmaceutical care issues and recommend interventions to medication related problems.

7. To help improve patient’s quality of life.

**C. SCOPE OF SERVICE**

1. The DMTAC service will operate in the clinic area during clinic day.

Subsequent visits shall be carried out in the pharmacy/ clinic area.

2. The DMTAC pharmacist will perform duties including assessing patients for pharmaceutical care issues, documenting actions and plans, providing appropriate education to patients, and completing follow-ups.

3. Activities at the clinic will be carried out according to the suggested workflow (Refer to Appendix 1, 2, and 3).

**D. MANPOWER**

DMTAC services can only be provided by trained pharmacists.

**E. APPOINTMENT**

All appointments will be scheduled by DMTAC pharmacists.

DMTAC PROTOCOL 3rd EDITION 2022 3


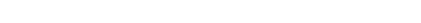

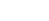

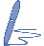


**F. OUTCOME MEASUREMENTS**


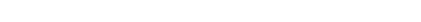

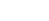

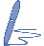

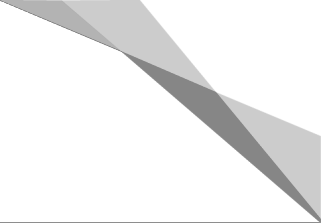


Every patient should be monitored and assessed during each DMTAC visit. All facilities providing DMTAC services shall monitor the following indicators as outcome measurements for the service:

(i) Medication adherence assessment (Refer to Appendix).

(ii) Glycaemic control, i.e. HbA1c, fasting plasma glucose (FPG) test, 2-hour post- prandial glucose (PPG) test, etc.

(iii) Medication knowledge i.e. DFIT score.

(iv) Other relevant monitoring parameters i.e. lipid profile, blood pressure, etc.

**G. WORKFLOW**

**1. PATIENT SELECTION**

Patient with diabetes currently managed in the Ministry of Health (MOH)

hospital or health clinic, with any of the following criteria:

1.1 Uncontrolled diabetes despite medication optimisation.

1.2 Non-adherent to medications (Refer to Appendix 5).

1.3 Failure to achieve individualised HbA1c targets (Refer to Table 1).

4 DMTAC PROTOCOL 3rd EDITION 2022

Table 1: Individualised HbA1c targets based on patient profile


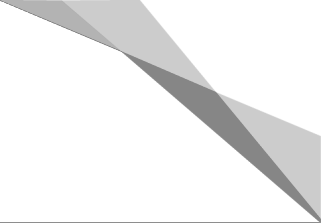


| **HbA1c**  **Target Descriptions** | |
| --- | --- |
| **≤ 6.5%**  **(Tight)** | • Newly and recently diagnosed (newly diagnosed is arbitrarily defined as T2DM < 1 year duration and recently diagnosed is defined as T2DM duration of < 5 years)  • Younger age  • Healthier  ➢ Long life expectancy, no cardiovascular disease (CVD) complications  • On medications that do not cause hypoglycaemia  • Low risk of hypoglycaemia |
| **6.6% - 7.0%** | All others |
| **7.1% - 8.0% (Less tight)** | • Elderly patients  • Presence of co-morbidities:  ➢ Advanced CVD  ➢ Coronary artery disease  ➢ Heart failure  ➢ Advanced renal failure (eGFR < 45ml/min/1.73m2)  ➢ Decompensated chronic liver disease  ➢ Dementia  ➢ Bed-bound i.e. stroke/ other co-morbidities  • Prone to/ experiencing severe hypoglycaemia  • Hypoglycaemia unawareness  • High risk of consequence of hypoglycaemia such as:  ➢ Those at risk of falling  ➢ Those who drive or operate machinery  • Those unlikely to benefit from strict glycaemic control  • Short life expectancy |

*Adapted with permission from “Ministry of Health; Malaysia (2020) "Clinical Practice Guidelines. Management of*

*Type 2 Diabetes Mellitus (6th Edition) p.43."*

1.4 Patient with co-morbidities or multiple medications

1.5 Patient with microvascular or macrovascular complications

1.6 Patient with frequent hypoglycaemia

1.7 Patient who has been discharged from DMTAC for at least six months can be re-recruited into DMTAC as a visit 1 patient*

**Reviewing a patient who was discharged less than six months shall only be considered as counselling.*

DMTAC PROTOCOL 3rd EDITION 2022 5


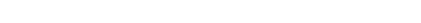

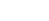

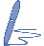

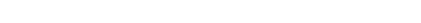

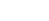

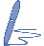


**2. INITIAL ASSESSMENT BY THE DMTAC PHARMACIST**

2.1 During the initial visit, the pharmacist will perform an initial assessment of the patient. The initial evaluation will involve:

2.1.1 Review of patient’s medical and medication history

2.1.2 Conducting a baseline assessment of: a) Past medical/ medication history b) Social/ family history

c) Occupational history (i.e. shift work, office work, student, etc.)

d) Patient’s knowledge on:

i. Diseases

ii. Medications

e) Device technique (insulin devices and/ or glucometers)

f) Medication adherence

g) Lifestyle (diet and physical activities)

h) Allergies history (drug, food, etc.)

2.1.3 Review of vital signs, laboratory parameters, and self-monitoring of blood glucose (SMBG) profiles

**Introduce and educate patients on SMBG (frequency and reporting)*

2.1.4 Determination of medication-related problems and issues

2.1.5 Patient (and/ or caregiver) interview

2.2 During the initial interview, the following will be discussed with the patient:

2.2.1 Objectives of DMTAC

2.2.2 Anticipated benefits to the patients and/ or caregivers

2.2.3 Treatment goals (i.e. individualised HbA1c goals, weight reduction target, etc.)

6 DMTAC PROTOCOL 3rd EDITION 2022

2.2.4 Patient’s specific drug therapy related needs

2.2.5 Patient’s rights and responsibilities in the program

2.3 Patient’s appointment book or prescription could be tagged as identification.

2.4 The pharmacist will then proceed with the **Education Modules for Diabetes Patients** (Appendix 4). The modules will be delivered at a pace based on patient’s understanding and knowledge assessment at every visit.

**3. SUBSEQUENT VISITS**

3.1 The subsequent visits shall be scheduled every 1-3 months, based on the patient’s need, his/ her current health status, other clinic visits, and medication refill appointments.

**Longer visit interval (more than 3 months) can be considered based on*

*DMTAC pharmacist’s discretion.*

3.2 All subsequent visits are preferably carried out at the clinic/ pharmacy (for patients attending DMTAC physically). If the phone or virtual sessions are considered, they should:

i. Be conducted every 1-3 months

ii. Include activities specified under subsections 3.3 to 3.6 iii. Be documented

iv. Have face-to-face review with DMTAC pharmacists when patients come for their scheduled clinic appointment with doctors

3.3 The pharmacist’s review at every visit includes :

i. Medication adherence assessment

ii. Glycaemic control review

iii. Review of relevant monitoring parameters

iv. Device technique assessment (insulin devices or glucometers)

v. Discussion of disease progression and complications

DMTAC PROTOCOL 3rd EDITION 2022 7


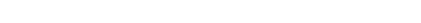

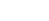

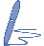


vi. Medication knowledge assessment


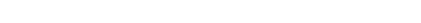

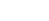

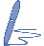

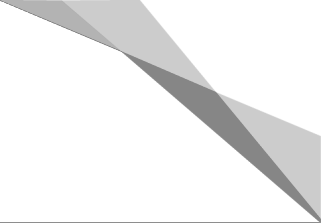


vii. Identification of treatment side effects

viii. Review and discussion of SMBG

ix. Lifestyle assessment

3.4 Educate patient using **Education Modules for Diabetes Patients**

(Appendix 4)

3.5 Conduct medication counselling

3.6 Adjust patient’s insulin doses and empower patient to do self-adjustment at home. Discussion with physician should be done if any concern arises

3.7 Make referral to other healthcare providers (if necessary)

3.8 Continue appointments until patient’s glycaemic and other laboratory parameters achieve target goals

**4. DMTAC PATIENTS REGISTRY**

A registry of DMTAC patients must be maintained.

**5. MISSED VISITS**

Patient who missed DMTAC visit will be seen on a new date that is convenient.

**6. PHARMACEUTICAL REVIEW**

6.1 Pharmaceutical care issue identification

a) Assess the patient and obtain all information required to identify any medication-related problem

6.2 Pharmaceutical care issue recommendation

a) Provide the most appropriate recommendation for the identified medication-related problem

8 DMTAC PROTOCOL 3rd EDITION 2022

b) Formulate a patient-specific action plan with the patient, including identifying specific therapeutic outcomes

c) Provide appropriate pharmacological and non-pharmacological recommendation to achieve the therapeutic outcomes

d) Take a holistic approach to patient care (i.e. consider the patient’s

medical, social, and financial needs) in establishing the action plan

6.3 Pharmaceutical care plan evaluation

a) Monitor patient’s adherence to the pharmaceutical care plan

b) Follow up on the patient’s progress to ensure the achievement of desired outcomes, making modifications to the existing plan if necessary

**7. MEDICATION DISPENSING AND COUNSELLING**

7.1 DMTAC pharmacist shall dispense the medications to the patient

(wherever feasible)

7.2 DMTAC pharmacist shall counsel the patient on medications

**8. DOCUMENTATION**

8.1 All the assessments and recommendations during DMTAC sessions should be documented in DMTAC notes electronically or manually

8.2 All DMTAC documentation should be available for viewing by doctors, DMTAC pharmacists and other healthcare professionals

8.3 All DMTAC notes should be kept for at least two years

DMTAC PROTOCOL 3rd EDITION 2022 9


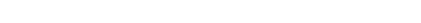

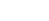

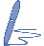

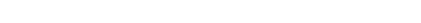

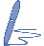

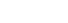


**9. DISCHARGE CRITERIA**

DMTAC pharmacists can discharge patients who fulfill any one of the following criteria:

a) Achieved individualised HbA1c targets for at least **two** consecutive readings.

b) Completed a minimum of **four visits**, with a good medication knowledge score (DFIT*) of 100% and good medication adherence.

c) Defaulted **six months** or **three** consecutive DMTAC visits.

d) Discharged or transferred to other facilities.

**DFIT: D=Dose, F=Frequency, I=Indication, T= Time of administration*

10 DMTAC PROTOCOL 3rd EDITION 2022

**H. REFERENCES**


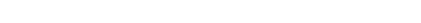

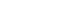

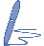


1. Alison C & Anselm S. (2020) “The effectiveness of diabetes medication therapy adherence clinic to improve glycaemic control among patients with type 2 diabetes mellitus: a randomised controlled trial”. The Medical Journal of Malaysia., **75**(3):246-253.

2. Hammad, M. A., Mohamed Noor, D. A., & Syed Sulaiman, S. A. (2017). “The effect of patients’ adherence on HbA1c control.” AMPSR: Archives of Medical and Pharmaceutical Sciences Research **1**(1):30-35.

3. Institute for Public Health (IPH), National Institutes of Health, Ministry of Health, Malaysia (2020). “National Health and Morbidity Survey (NHMS) 2019: Vol. I: NCDs – Non- Communicable Diseases: Risk Factors and other Health Problems.” **1**:1-392.

4. International Diabetes Federation. IDF Diabetes Atlas, 10th edn. Brussels, Belgium: International Diabetes Federation, 2021.

5. Korcegez EI, Sancar M, Demirkan K (2017). “Effect of a pharmacist-led program on improving outcomes in patients with type 2 diabetes mellitus from Northern Cyprus: A randomized controlled trial.” Journal of Managed Care & Specialty Pharmacy **23**(5):573-582.

6. Krass, I, C. L., Armour, B., Mitchell, M., Brillant, R., Dienaar, J., Hughes, P., Lau, G., Peterson, K., Stewart, S., Taylor & J. Wilkinson. (2006). “The Pharmacy Diabetes Care Program: assessment of a community pharmacy diabetes service model in Australia”. Diabetic Medicine. **24**(6), 677-683.

7. You, L.X., Selvadurai, S., Yee, C.K., Noh, N.B., Bao, G.C., Joyce, T., Hamdi, A.H. & Haron, N. (2015). “Impact of Pharmacist-Managed Diabetes Medication Adherence Clinic (DMTAC) in Government Health Clinics”. Malaysian Journal of Pharmaceutical Sciences, **13**(1), 43.

8. Lim P.C. & Lim K. (2010). “Evaluation of a pharmacist-managed diabetes medication therapy adherence clinic”. Pharmacy Practice. **8**(4):250-4.

9. Lim, P. C., K. Lim, Z. C. Embee, M. A. Hassali, A. Thiagarajan & T. M. Khan (2016). "Study investigating the impact of pharmacist involvement on the outcomes of diabetes medication therapy adherence program Malaysia." Pakistan Journal of Pharmaceutical Sciences **29**(2):

595-601.

10. Loganadan NK, Lim KY, Nur NM, & Ariffin F (2012). "Cost-effectiveness of pharmacist managed medication therapy adherence clinic (MTAC) on type 2 diabetes patients in a tertiary hospital in Malaysia." Pharmacotherapy **32**(10): pp. E270-E270.

11. Ministry of Health; Malaysia (2020) "Clinical practice guidelines. management of type 2 diabetes mellitus (6th Edition)."

12. Mohamad, N., Che Embee, Z., Lim, P.C., Chong , L.Y., Ting, A.S.H., Nasir, N.M., et al (2010).

A Multicenter Study on the Outcome of Pharmacist Managed Diabetes Medication Therapy

Adherence Clinic (DMTAC) in Malaysia. Diabetes Asia Confernce 2010 Abstract Book, 69

13. Rothman, R. L., R. Malone, B. Bryant, A. K. Shintani, B. Crigler, D. A. Dewalt, R. S. Dittus, M. Weinberger & M. P. Pignone (2005). "A randomized trial of a primary care-based disease management program to improve cardiovascular risk factors and glycated hemoglobin levels in patients with diabetes." American Journal of Medicine **118**(3): 276-284.

14. Rozenfeld, Y., Hunt, J. S., Plauschinat, C., & Wong, K. S. (2008). “Oral Antidiabetic Medication Adherence and Glycemic Control”. The American Journal of Managed Care.14:71-75

15. Stratton, I. M., A. I. Adler, H. A. Neil, D. R. Matthews, S. E. Manley, C. A. Cull, D. Hadden, R.

C. Turner & R. R. Holman (2000). "Association of glycaemia with macrovascular and microvascular complications of type 2 diabetes (UKPDS 35): Prospective observational study." British Medical Journal **321**(7258): 405-412.

16. Tai C.W. (2016) An evaluation on pharmacist-managed diabetes medication therapy adherence clinic (DMTAC) in primary health clinic of Johor Bahru district, Malaysia. Johor Health Journal, 12:41-53.

DMTAC PROTOCOL 3rd EDITION 2022 11

**I. APPENDICES**


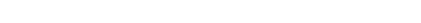

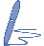

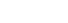


**Appendix 1:**

DMTAC Workflow (First Visit)

**Appendix 2:**

DMTAC Workflow (Subsequent Visit)

**Appendix 3:**

DMTAC Workflow (Phone/ Virtual Call Visit)

**Appendix 4:**

Education Modules for Diabetes Patients

**Appendix 5:**

Malaysia Medication Adherence Assessment Tool (MyMAAT)

**Appendix 6:**

PhIS Documentation

**Appendix 7:**

DMTAC Pharmacotherapy Review Form

**Appendix 8:**

DMTAC Session Checklists

12 DMTAC PROTOCOL 3rd EDITION 2022

*APPENDIX 1*

**DIABETES MEDICATION THERAPY ADHERENCE CLINIC WORKFLOW**

**FIRST DMTAC VISIT**

NURSE

PHARMACIST

REGISTRATION

RECRUITMENT & BASELINE ASSESSMENT

• Past medical/ medication history

• Allergies history (food & drug)

• Social/ family history

• Occupational history

• Knowledge on disease, medications

• Device knowledge

• Medication adherence

• Lifestyle (Diet & physical activities)

PHARMACIST

REVIEW & RECOMMENDATIONS

• Vital signs, lab parameters & SMBG

• Pharmaceutical Care Issues

• Goals & targets

PHARMACIST

COUNSELLING & EDUCATIONS

*Based on patient’s understanding & needs during visit –

| REVIEW & TREATMENT | |
| --- | --- |
|  |  |
| MEDICATION DISPENSING AND COUNSELLING (wherever feasible) | |
|  |  |

**APPENDIX 4**

DOCTOR

PHARMACIST

PHARMACIST

SCHEDULE FOR NEXT VISIT

(*To obtain patients’ consent if they agreed for Virtual

Call Visits)

DOCUMENTATION

DMTAC PROTOCOL 3rd EDITION 2022 13


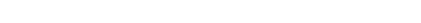

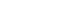

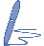


**SUBSEQUENT VISIT (Every 1–3 months)**

PHARMACIST

PHARMACIST

TRACE PATIENT’S RECORDS

PHARMACIST REVIEW

• Medication adherence assessment

• Glycaemic control review

• Review of relevant monitoring parameters

• Device technique assessment (insulin

devices or glucometers)

• Discussion of disease progression and complications

• Medication knowledge assessment

• Identification of treatment side effects

• Review and discussion of SMBG

• Lifestyle assessment

PHARMACIST

PHARMACIST

| MEDICATION REFILL, DISPENSING AND COUNSELLING  (wherever feasible) | |
| --- | --- |
|  |  |
| SCHEDULE FOR NEXT VISIT  (*To obtain patients’ consent if they agreed for Virtual  Call Visits) | |
|  |  |

PHARMACIST

PHARMACIST


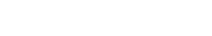


REINFORCEMENT, COUNSELLING & EDUCATION

*Based on patient’s understanding & needs during visit –

**APPENDIX 4**


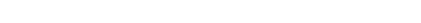

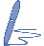

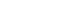


14 DMTAC PROTOCOL 3rd EDITION 2022

**PHONE/ VIRTUAL CALL VISITS (Every 1-3 months)**

**Location: Diabetes Clinic**

PHARMACIST

PHARMACIST

TRACE PATIENT’S RECORDS

ASSESSMENT AND REVIEW

• Medication adherence assessment

• Glycaemic control review

• Review of relevant monitoring parameters

• Device technique assessment (insulin devices or glucometers)

• Discussion of disease progression and complications

• Medication knowledge assessment

• Identification of treatment side effects

• Review and discussion of SMBG

• Lifestyles assessment

PHARMACIST

PHARMACIST

PHARMACIST

REINFORCEMENT, COUNSELLING & EDUCATION

*Based on patient’s understanding &

needs during visit – **APPENDIX 4**

SCHEDULE FOR NEXT PHONE / VIRTUAL CALL VISIT / FACE-TO-FACE VISIT

DOCUMENTATION

DMTAC PROTOCOL 3rd EDITION 2022 15


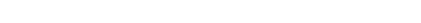

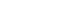

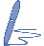

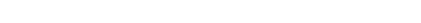

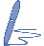

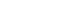


**EDUCATION MODULES FOR DIABETES PATIENTS**

**First Module**

❑ Brief overview of diabetes (symptoms, complications, etc)

❑ Therapeutic goals, specifically blood glucose (HbA1c, FPG, etc.)

❑ Specific discussion on medication use/adverse effects with the patient

❑ Medication storage at home

❑ Patient’s issues / concerns

**Second Module**

❑ Specific discussion on insulin use/adverse effects with the patient

❑ Reassess insulin injection technique & storage

❑ Signs and symptoms of hypoglycemia or hyperglycemia

❑ Self-monitoring of blood glucose (SMBG) - how, when, why, etc. (if applicable)

❑ Special instructions: Sick day management (if any) and Ramadan/ fasting month medication dose adjustment

❑ Patient’s issues / concerns

**Third Module**

❑ Benefits of exercise

❑ Introduction to basic nutrition (including basic carbohydrate counting)

❑ Benefits of quit smoking

❑ Patient’s issues / concerns

**Fourth Module**

❑ In-depth discussion on complications in diabetes (macrovascular & microvascular complications)

❑ Prevention, detection, and treatment of complications: review of targets for cholesterol and blood pressure

❑ Discussion on medication use and adverse effects with the patient (anti- hypertensives and anti-cholesterol)

❑ Foot care

❑ Patient’s issues / concerns

16 DMTAC PROTOCOL 3rd EDITION 2022


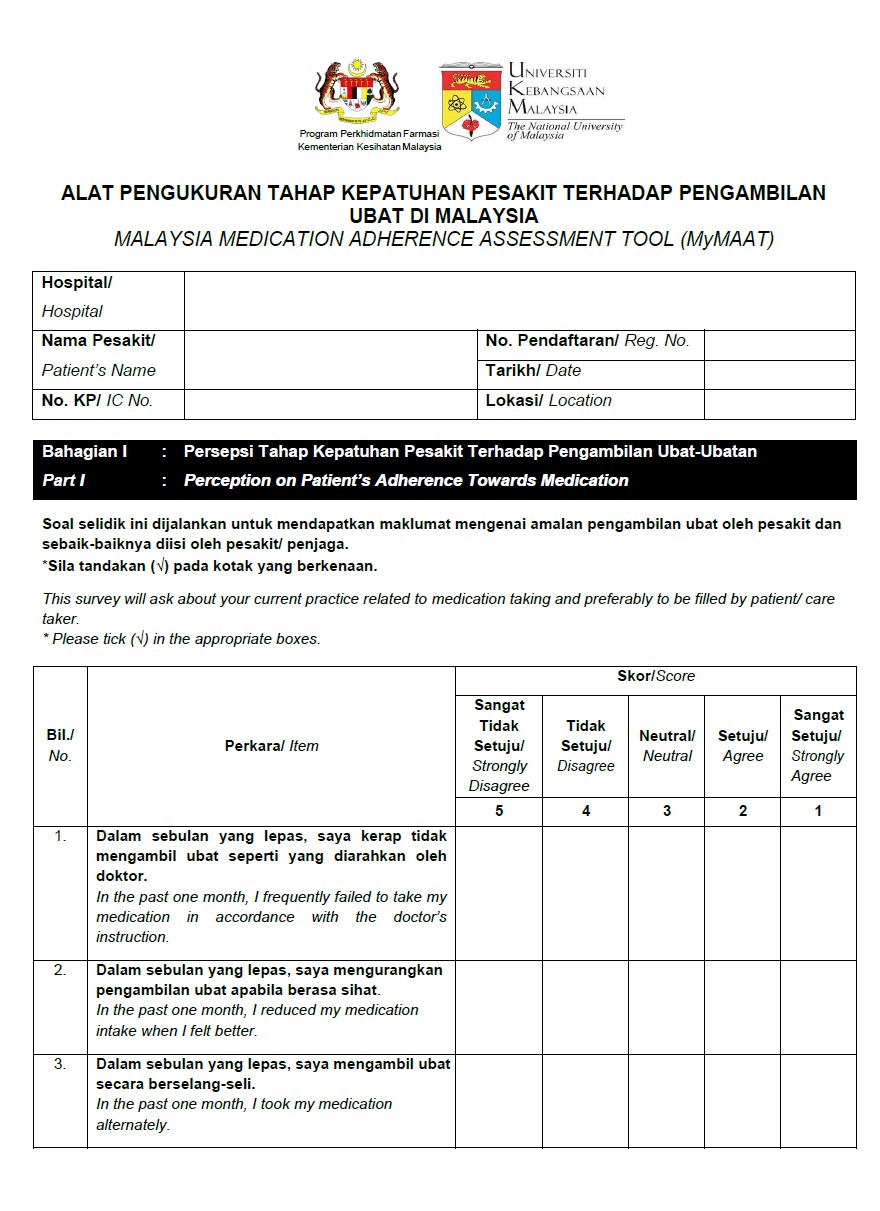


DMTAC PROTOCOL 3rd EDITION 2022 17


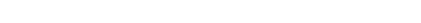

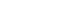

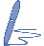

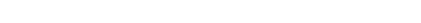

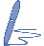

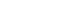

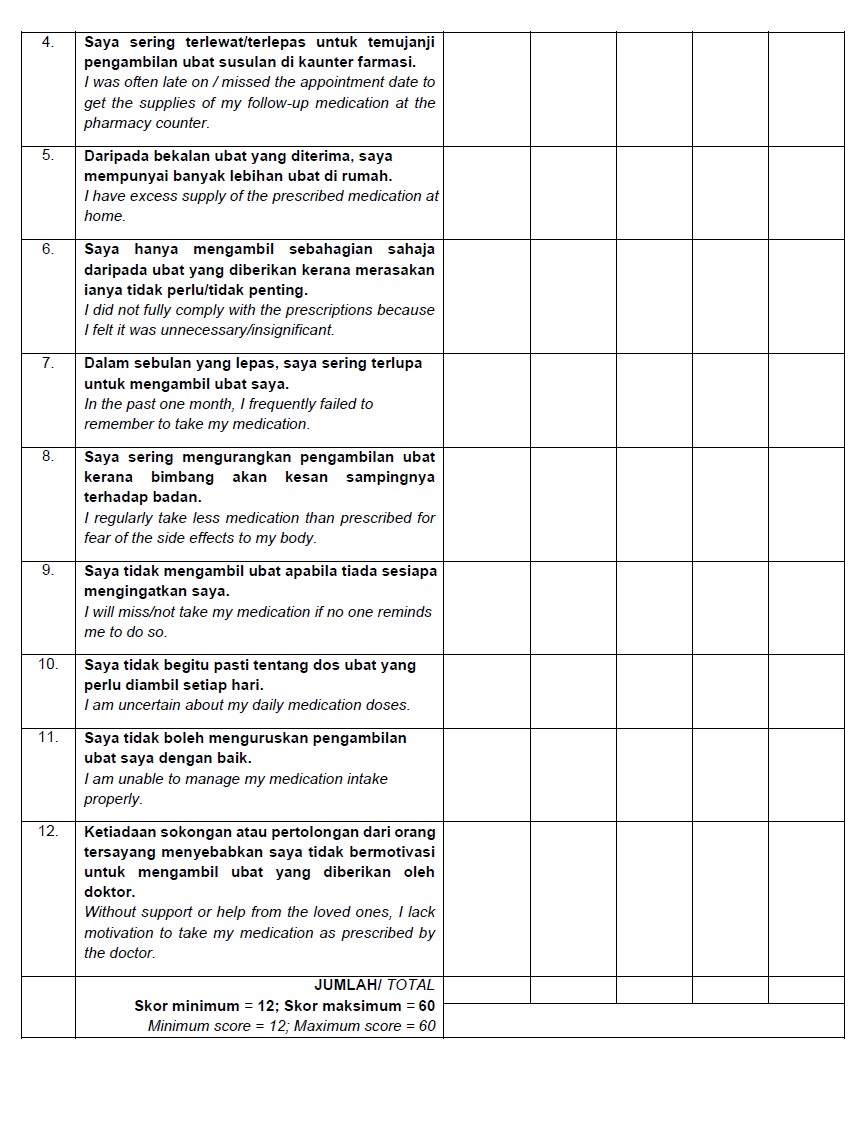


18 DMTAC PROTOCOL 3rd EDITION 2022


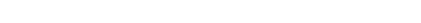

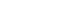

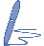

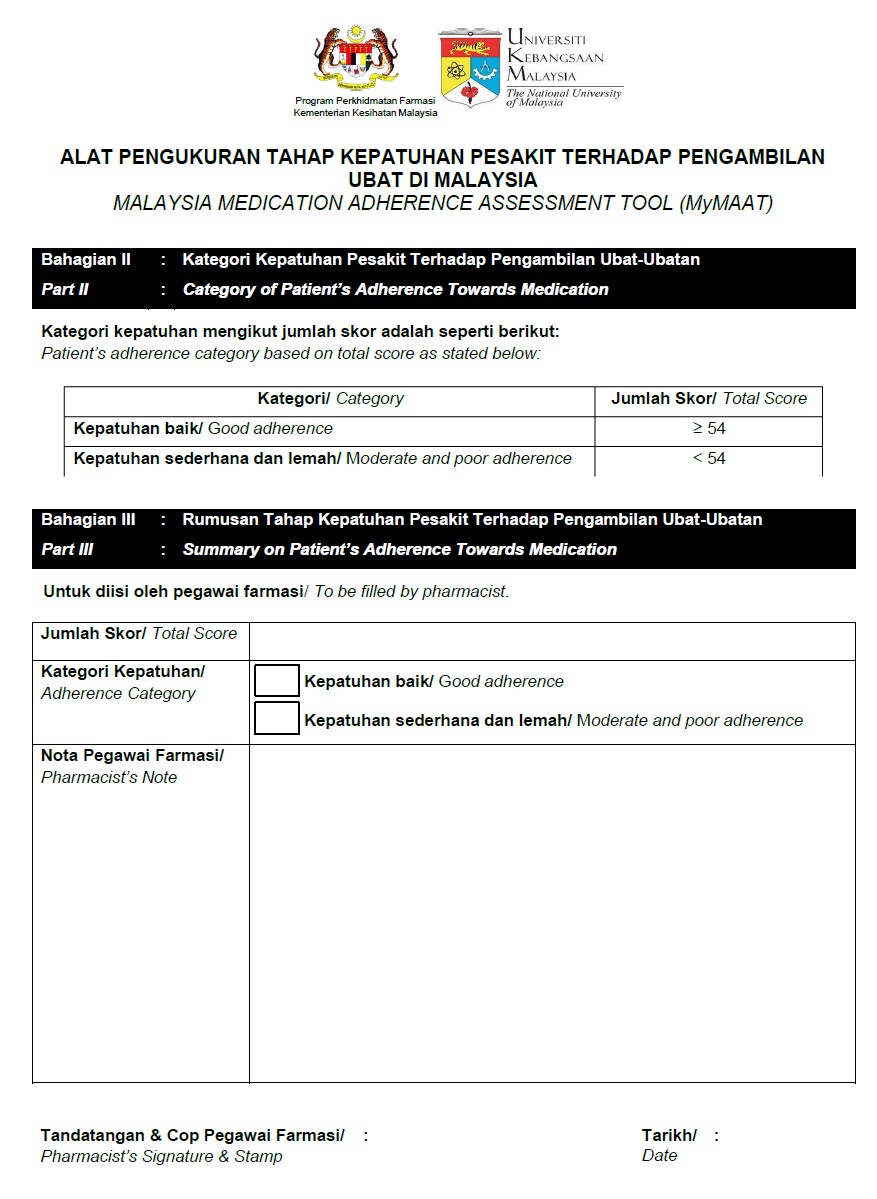


DMTAC PROTOCOL 3rd EDITION 2022 19

*APPENDIX 6*


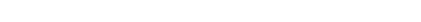


*PhIS : Social History*

20 DMTAC PROTOCOL 3rd EDITION 2022

DMTAC PROTOCOL 3rd EDITION 2022 21

22 DMTAC PROTOCOL 3rd EDITION 2022

DMTAC PROTOCOL 3rd EDITION 2022 23

24 DMTAC PROTOCOL 3rd EDITION 2022

DMTAC PROTOCOL 3rd EDITION 2022 25

26 DMTAC PROTOCOL 3rd EDITION 2022

DMTAC PROTOCOL 3rd EDITION 2022 27

28 DMTAC PROTOCOL 3rd EDITION 2022

DMTAC PROTOCOL 3rd EDITION 2022 29

*APPENDIX 7*

**DIABETES MEDICATION THERAPY ADHERENCE CLINIC PHARMACOTHERAPY REVIEW**

**Pharmacy Department:**

Name:**………………………………………………………...** I/C:**………………………………….** Age:**…………………………..** Gender: M / F RN:**…………………………………** Race:**……………………………………..** Contact Number: **…………………………………...**

Date of visits:**…………………../…………………../…………………../…………………../………………..**

Past Medical History (summary):

Social/Family History:

Smoking:

Alcohol:

Drug Allergies:

Diet and Lifestyle

Medication Lists (Before enrolment)

1. 8.

2. 9.

3. 10.

4. 11.

5. 12.

6. 13.

7. 14.

30 DMTAC PROTOCOL 3rd EDITION 2022

| **Review of Patient’s Understanding of Medication (Primarily OGLDs)** | | | | |
| --- | --- | --- | --- | --- |
| **Medication** | **Visit 1** | **Visit 2** | **Visit 3** | **Visit 4** |
|  | **D F I T** | **D F I T** | **D F I T** | **D F I T** |
|  |  |  |  |  |
|  |  |  |  |  |
|  |  |  |  |  |
|  |  |  |  |  |
|  |  |  |  |  |
|  |  |  |  |  |
|  |  |  |  |  |
|  |  |  |  |  |
|  |  |  |  |  |
|  |  |  |  |  |
|  |  |  |  |  |
|  |  |  |  |  |
|  |  |  |  |  |
|  |  |  |  |  |
| **Score (%)** |  |  |  |  |
| **Review of Patient’s Understanding of Medication (Primarily OGLDs)** | | | | |
| **Medication** | **Visit 5** | **Visit 6** | **Visit 7** | **Visit 8** |
|  | **D F I T** | **D F I T** | **D F I T** | **D F I T** |
|  |  |  |  |  |
|  |  |  |  |  |
|  |  |  |  |  |
|  |  |  |  |  |
|  |  |  |  |  |
|  |  |  |  |  |
|  |  |  |  |  |
|  |  |  |  |  |
|  |  |  |  |  |
|  |  |  |  |  |
|  |  |  |  |  |
|  |  |  |  |  |
|  |  |  |  |  |
|  |  |  |  |  |
| **Score (%)** |  |  |  |  |

Key:

D = Dose F = Frequency I = Indication T = Time of administration

Pharmacist’s Notes:

DMTAC PROTOCOL 3rd EDITION 2022 31

| **Parameters** | **Normal**  **Value** | **Date** | | | | | | | | | | | | |
| --- | --- | --- | --- | --- | --- | --- | --- | --- | --- | --- | --- | --- | --- | --- |
|  |  |  |  |  |  |  |  |  |  |  |  |  |  |  |
| **GLYCAEMIC CONTROL** | | | | | | | | | | | | | | |
| FPG (mmol/L) | 4.4-7.0 |  |  |  |  |  |  |  |  |  |  |  |  |  |
| 2HPP (mmol/L) | 4.4-8.5 |  |  |  |  |  |  |  |  |  |  |  |  |  |
| RPG (mmol/L) | <10.0 |  |  |  |  |  |  |  |  |  |  |  |  |  |
| HbA1c (%) | <7.0%  (for most) Individualised* |  |  |  |  |  |  |  |  |  |  |  |  |  |
| **PHYSICAL PARAMETERS** | | | | | | | | | | | | | | |
| Blood Pressure  (mmHg) | ≤130-139 /  70-79 |  |  |  |  |  |  |  |  |  |  |  |  |  |
| Weight (kg) |  |  |  |  |  |  |  |  |  |  |  |  |  |  |
| Height (cm) |  |  |  |  |  |  |  |  |  |  |  |  |  |  |
| Waist circumference | M <90cm, F < 85cm |  |  |  |  |  |  |  |  |  |  |  |  |  |
| BMI | <23kg/m2 |  |  |  |  |  |  |  |  |  |  |  |  |  |
| **RENAL PROFILE** | | | | | | | | | | | | | | |
| Na (mmol/L) | 135-145 |  |  |  |  |  |  |  |  |  |  |  |  |  |
| K (mmol/L) | 3.5-5.0 |  |  |  |  |  |  |  |  |  |  |  |  |  |
| Urea (mmol/L) | 2.8-7.2 |  |  |  |  |  |  |  |  |  |  |  |  |  |
| Sr Creatinine  (µmol/L) | 57-130 |  |  |  |  |  |  |  |  |  |  |  |  |  |
| eGFR (ml/min) |  |  |  |  |  |  |  |  |  |  |  |  |  |  |
| Urine Protein |  |  |  |  |  |  |  |  |  |  |  |  |  |  |

32 DMTAC PROTOCOL 3rd EDITION 2022

| **Parameters** | **Normal**  **Value** | **Date** | | | | | | | | | | | | |
| --- | --- | --- | --- | --- | --- | --- | --- | --- | --- | --- | --- | --- | --- | --- |
|  |  |  |  |  |  |  |  |  |  |  |  |  |  |  |
| **LIVER FUNCTION** | | | | | | | | | | | | | | |
| Total Protein (g/L) | 66-87 |  |  |  |  |  |  |  |  |  |  |  |  |  |
| Albumin (g/L) | 35-52 |  |  |  |  |  |  |  |  |  |  |  |  |  |
| Globulin (g/L) | 20-36 |  |  |  |  |  |  |  |  |  |  |  |  |  |
| Total Bilirubin  (µmol/L) | 0-24 |  |  |  |  |  |  |  |  |  |  |  |  |  |
| AST (IU/L) | <32 |  |  |  |  |  |  |  |  |  |  |  |  |  |
| ALT (IU/L) | <41 |  |  |  |  |  |  |  |  |  |  |  |  |  |
| ALP (IU/L) (>15yrs)  (3-15yrs) | 34-104  98-369 |  |  |  |  |  |  |  |  |  |  |  |  |  |
| **LIPID PROFILE** | | | | | | | | | | | | | | |
| T. Cholesterol/TC (mmol/L) | 3.5-5.7 |  |  |  |  |  |  |  |  |  |  |  |  |  |
| Triglyceride/TG (mmol/L) | ≤1.7 |  |  |  |  |  |  |  |  |  |  |  |  |  |
| LDL-C (mmol/L) | ≤2.6 |  |  |  |  |  |  |  |  |  |  |  |  |  |
| HDL-C (mmol/L) | Male: >1.0  Female:>1.2 |  |  |  |  |  |  |  |  |  |  |  |  |  |
| **OTHERS** | | | | | | | | | | | | | | |
|  |  |  |  |  |  |  |  |  |  |  |  |  |  |  |
|  |  |  |  |  |  |  |  |  |  |  |  |  |  |  |
|  |  |  |  |  |  |  |  |  |  |  |  |  |  |  |
|  |  |  |  |  |  |  |  |  |  |  |  |  |  |  |

DMTAC PROTOCOL 3rd EDITION 2022 33

**Visit:**...............................................................................................................................................

**PHARMACEUTICAL**

**CARE ISSUES INTERVENTION OUTCOME**

34 DMTAC PROTOCOL 3rd EDITION 2022

| **No. Aspect** |
| --- |
| **1** Introduce yourself – The purpose of DMTAC |
| **2** Disease knowledge assessment |
| **3** A brief overview of diabetes |
| **4** Educate patients by using DMTAC Education Modules |
| **5** Therapeutic goals (i.e. HbA1c, FPG, Lipid, BP, Weight) |
| **6** Hyperglycemia and hypoglycemia symptoms |
| DM complications assessment and education  **7** ~ summarise patient’s DM complications (diabetic kidney disease, retinopathy, cardiovascular disease, diabetic foot problem/prior amputation, erectile  dysfunction, neuropathy, etc.) |
| Discussion of medication(s) used  **8** ~ either OGLDs*/insulin (emphasize the need for insulin in the future)  ~ use of cardiorenal protective medications (i.e. statin, RAAS inhibitor,  antiplatelet, SGLT2i, etc.) |
| **9** Discussion of any adverse effects with patients (with oral medication or insulin) |
| Diet and lifestyle  **10** ~ Meals for breakfast/lunch/dinner  ~ Exercise/smoking/alcohol |
| **11** Self-monitoring blood glucose - how, when, where  ~ Give booklet, emphasise the need for SMBG |
| **12** Sick day management & course of action to be taken |
| Discuss laboratory parameters  **13** ~ FPG/ HbA1c/ lipid profile/ LFT*/ BP/ SMBG/ renal profile/ urine albumin/ protein / UACR |
| **14** Discuss dose/timing/technique/site of injection/ needle use |
| **15** Discuss storage of insulin |
| **16** Discuss frequency to change needles/ disposal of needles |
| **17** Assess medication adherence score |
| **18** Assess understanding of medications – DFIT |
| **19** Review and set individualised patient targets |
| **20** Discuss insulin dose adjustment (if any) |
| **21** Discuss changes in medications (if any) |
| **22** Other counselling (if any) |
| **23** Get a new appointment for pharmacist review |

***This checklist serves as a reference only. Each DMTAC session shall be carried out*

*based on individual needs.*

DMTAC PROTOCOL 3rd EDITION 2022 35

| **2HPP** | 2 hours post-prandial |
| --- | --- |
| **ALP** | Alkaline phosphatase |
| **ALT** | Alanine aminotransferase |
| **AST** | Aspartate transaminase |
| **BMI** | Body mass index |
| **BP** | Blood pressure |
| **CVD** | Cardiovascular disease |
| **DFIT** | Dose, Frequency, Indication, Time of administration |
| **DM** | Diabetes Mellitus |
| **DMTAC** | Diabetes Medication Therapy Adherence Clinic |
| **eGFR** | Estimated glomerular filtration rate |
| **FPG** | Fasting plasma glucose |
| **HbA1c** | Glycosylated haemoglobin |
| **HDL-C** | High-density lipoprotein - Cholesterol |
| **LDL-C** | Low-density lipoprotein - Cholesterol |
| **LFT** | Liver function test |
| **MOH** | Ministry of Health |
| **MyMAAT** | Malaysia Medication Adherence Assessment Tool |
| **NHMS** | National Health and Morbidity Survey |
| **OGLD** | Oral glucose lowering drug |
| **PhIS** | Pharmacy Information Systems |
| **PPG** | Post-prandial glucose |
| **RAAS** | Renin-angiotensin-aldosterone system |
| **RPG** | Random plasma glucose |
| **SGLT2i** | Sodium glucose cotransporter-2 inhibitor |
| **SMBG** | Self-monitoring blood glucose |
| **T2DM** | Type 2 diabetes mellitus |
| **TC** | Total cholesterol |
| **TG** | Triglyceride |
| **UACR** | Urine albumin-creatinine ratio |
| **UKPDS** | United Kingdom Prospective Diabetes Study |

36 DMTAC PROTOCOL 3rd EDITION 2022

**DIABETES MEDICATION THERAPY ADHERENCE PROTOCOL**

**PHARMACEUTICAL SERVICES PROGRAM MINISTRY OF HEALTH, MALAYSIA**

**LOT 36, JALAN PROFESOR DIRAJA UNGKU AZIZ,**

**46200 PETALING JAYA, SELANGOR, MALAYSIA**

**TEL: 603-7841 3200**

**WEBSITE**[**: WWW.PHARMACY.GOV.MY**](http://WWW.PHARMACY.GOV.MY)
